# Alpinetin Suppresses Zika Virus-Induced Interleukin-1β Production and Secretion in Human Macrophages

**DOI:** 10.3390/pharmaceutics14122800

**Published:** 2022-12-14

**Authors:** Nitwara Wikan, Saranyapin Potikanond, Phateep Hankittichai, Phatarawat Thaklaewphan, Sathit Monkaew, Duncan R. Smith, Wutigri Nimlamool

**Affiliations:** 1 Department of Pharmacology, Faculty of Medicine, Chiang Mai University, Chiang Mai 50200, Thailand; 2 Institute of Molecular Biosciences, Mahidol University, Salaya, Nakhon Pathom 73170, Thailand; 3 Research Center for Development of Local Lanna Rice and Rice Products, Chiang Mai University, Chiang Mai 50200, Thailand

**Keywords:** Zika virus, alpinetin, inflammation, IL-1β, p38 MAPK

## Abstract

Zika virus (ZIKV) infection has been recognized to cause adverse sequelae in the developing fetus. Specially, this virus activates the excessive release of IL-1β causing inflammation and altered physiological functions in multiple organs. Although many attempts have been invested to develop vaccine, antiviral, and antibody therapies, development of agents focusing on limiting ZIKV-induced IL-1β release have not gained much attention. We aimed to study the effects of alpinetin (AP) on IL-1β production in human macrophage upon exposure to ZIKV. Our study demonstrated that ZIKV stimulated IL-1β release in the culture supernatant of ZIKV-infected cells, and AP could effectively reduce the level of this cytokine. AP exhibited no virucidal activities against ZIKV nor caused alteration in viral production. Instead, AP greatly inhibited intracellular IL-1β synthesis. Surprisingly, this compound did not inhibit ZIKV-induced activation of NF-κB and its nuclear translocation. However, AP could significantly inhibit ZIKV-induced p38 MAPK activation without affecting the phosphorylation status of ERK1/2 and JNK. These observations suggest the possibility that AP may reduce IL-1β production, in part, through suppressing p38 MAPK signaling. Our current study sheds light on the possibility of using AP as an alternative agent for treating complications caused by ZIKV infection-induced IL-1β secretion.

## 1. Introduction

Zika virus infection can be asymptomatic or may cause mild symptoms [1]. Nevertheless, it may cause severe diseases including congenital brain abnormalities [2,3] and Guillain–Barré syndrome [2,4,5]. In addition, lack of symptoms in ZIKV-infected cases may also lead to potential complications. For instance, it has been reported that the incidence of ZIKV-associated birth abnormalities (primarily brain abnormalities and microcephaly) of fetuses or infants born to asymptomatic or symptomatic women with ZIKV infection was not different [6]. Besides targeting important cells residing in the brain such as neural progenitor cells, astrocytes, and functional neurons, ZIKV can also infect cells in many organs [7]. Specifically, it has been demonstrated that ZIKV infected eyes, vagina, placenta, uterus, and testis [8,9]. ZIKV could also target renal proximal tubular epithelia and glomerular parenchymal cells [10,11]. Moreover, ZIKV infected monocytes and macrophages and utilized these cells as its replication reservoir [12,13].

In response to ZIKV infection, interleukin-1β (IL-1β) was highly increased, suggesting that it may be the cause of the ZIKV-associated diseases [14]. Usually, IL-1β secretion mediates the host defense system against pathogens. For example, IL-1β was proved to regulate the generation of cholesterol to 25-hydroxycholesterol by the enzyme cholesterol 25-hydroxylase (CH25H), and that exhibited broad antiviral properties [15]. Nevertheless, an excessive level of IL-1β upon infection by various viruses may lead to various inflammatory diseases, including hepatitis and encephalitis [16,17,18]. Particularly, one study reported that patients or mice with ZIKV infection exhibited high levels of IL-1β in the serum [19]. The same study also proved that ZIKV infected human peripheral blood mononuclear cells (PBMCs), TPA-activated macrophages, and bone marrow-derived dendritic cells (BMDCs) and stimulated IL-1β production (through activation of pathogen-associated molecular patterns (PAMPs)) and IL-1β maturation (through stimulation of NLRP3 inflammasome activated by ZIKV NS5 protein) [19]. Another team verified these findings and provided additional data that the intracellular production of reactive oxygen species (ROS) is crucial for NS5-induced NLRP3 inflammasome activation during ZIKV infection [20]. The presence of IL-1β in the serum of patients infected with ZIKV suggests that IL-1β enters the circulation and may trigger IL-1 receptors on many different cell types throughout the body. It is reasonable that this circulating IL-1β may give rise to many systemic inflammatory diseases. For example, it was disclosed that ZIKV stimulated IL-1β generation in the kidney and induced renal cell apoptosis, which consequently decreased aquaporin expression, leading to water reabsorption disorder [21]. Hence, agents that can inhibit the production or maturation of IL-1β in response to ZIKV infection may help reduce IL-1β–mediated diseases [22]. This statement can be supported by a previous study reporting that placental ZIKV infection induced IL-1β secretion, which provoked perinatal developmental abnormalities, and IL-1 receptor antagonist treatment alleviated ZIKV-induced placental dysfunction and perinatal injury [23].

The current study aimed to investigate the effects of alpinetin (AP), a natural flavone (7-Hydroxy-5-methoxyflavanone) prevalent in many medicinal plants in the ginger family (Zingiberaceae), on the ZIKV-induced production and secretion of IL-1β in human macrophages. Our focus was based on previous data that this compound exhibited anti-inflammatory activities in various models [24]. Specifically, AP could strongly downregulate the expression of IL-1β in RAW 264.7 macrophages induced with lipopolysaccharide (LPS) and inhibited LPS-stimulated acute lung injury in animal models [25]. Moreover, AP diminished inflammation in TPA-activated THP-1 macrophage and dextran sulfate sodium (DSS)-induced acute colitis models by inhibiting NF-κB and NLRP3 inflammasome sensitization [26]. An additional investigation revealed that AP markedly stimulated PPAR-γ and mitigated LPS-induced inflammation in THP-1 cells through reducing ERK, JNK, and p38 MAPK activation [27]. Furthermore, AP was able to decrease oxidative stress and inflammation in ulcerative colitis mice [28]. However, the effect of AP on modulating ZIKV-induced production and the release of IL-1β in human macrophages is unknown. Our study provides promising data that AP strongly suppresses the production of IL-1β in ZIKV-infected THP-1 macrophages, at least in part through inhibiting p38 MAPK phosphorylation. These findings offer new insights into the mechanism of AP in controlling ZIKV-induced inflammation and may be developed as an alternative therapeutic option for ZIKV-associated diseases.

## 2. Materials and Methods

### 2.1. Cell Culture and Virus

The current study utilized 2 cell lines, which included THP-1 (ATCC, Number TIB-202) and Vero (ATCC, Number CCL-81) (ATCC, Manassas, VA, USA). THP-1 cells were maintained in RPMI-1640 medium (Thermo Fisher Scientific, Waltham, MA, USA), to which was added FBS (10%) (Merck KGaA, Darmstadt, Germany), penicillin (100 U/mL), and streptomycin sulfate (100 μg/mL) (Thermo Fisher Scientific, Waltham, MA, USA). Vero cells were maintained in Dulbecco’s modified Eagle’s medium (DMEM) (Merck KGaA, Darmstadt, Germany) with supplements similar to those for THP-1 cells. Both cell types were cultured in an incubator set at 37 °C, 5% CO_2_. ZIKV used in this study was ZIKV (MU1-2017) [29].

### 2.2. Stimulation of THP-1 Cell Differentiation

THP-1 cells were plated and differentiated to macrophages in 24-well plates by using 60 nM of 12-O-Tetradecanoylphorbol-13-acetate (TPA) (Catalog number (Cat No.) J63910) (Thermo Fisher Scientific, Waltham, MA, USA) in RPMI complete media for 14 h. Media were changed to TPA-free RPMI complete media, and cells were cultured for additional 24 h. After 24 h, THP-1 macrophages were ready for infection with ZIKV and treatment with AP (Chengdu Biopurify Phytochemicals Ltd., Chengdu, China). The chemical structure and its mass were presented in Supplementary Table S1.

### 2.3. ZIKV Infection and AP Treatment

For experiments aimed to test the inhibitory effects of AP on IL-1β release in the culture supernatants of THP-1 cells infected with ZIKV by ELISA and Western blotting, the cells were induced with 60 µM of TPA and seeded on 24-well plates (3 × 10^5^ cells/well) overnight. Media were changed to complete media, and cells were cultured in standard conditions for 1 day. TPA-induced THP-1 cells were then infected with ZIKV at multiplicity of infection (MOI) of 5 for 2 h. After infection, the cells were treated with AP or DMSO at various concentrations and incubated for 48 h under standard conditions. Each condition was performed in independent triplicate. Supernatants were collected to determine the level of IL-1β by ELISA or kept at −80 °C to determine infectious virions. The cells attaching to the plate surface were lysed with lysis buffer and kept at −20 °C for Western blot analysis. For experiments aimed to detect the effects of AP on intracellular production of IL-1β, ZIKV E protein, and ZIKV NS5 protein, THP-1 cells were differentiated and seeded on 24-well plate (3 × 10^5^ cells/well) overnight followed by the replacement of complete media for 1 day. TPA-induced THP-1 were pretreated with AP or DMSO at various concentrations for 3 h followed by ZIKV infection. Each condition was performed in independent triplicate. Cells were lysed and collected at various time periods for Western blot analysis.

### 2.4. MTT Assay

THP-1 macrophages (1 × 10^5^ cells/well) seeded and differentiated in 96-well plates were treated with AP at various concentrations (0 to 400 μM), in RPMI complete media for 48 h. DMSO (0 to 0.5400%) was used as a vehicle control. After 48 h of treatment, cells were gently washed 1 time with phosphate buffer saline (PBS) (Thermo Fisher Scientific, Waltham, MA, USA) prior to the addition of the RPMI complete media (200 μL) with 0.5 mg/mL of MTT reagent (Thermo Fisher Scientific, Waltham, MA, USA). After 1 h, the MTT-containing supernatant in each well was discarded, and the cells were lysed with 100 μL of DMSO. The developed color was detected with a plate reader (BioTek Instruments, Winooski, VT, USA) set for detection at 570 nm.

### 2.5. Enzyme-Linked Immunosorbent Assay (ELISA)

IL-1β level in the supernatants of the control and treatment groups was evaluated by the human IL-1β DuoSet ELISA Kit (Cat No. DY201) (R&D Systems, Inc., Minneapolis, MN, USA) following the specific protocol instructed by the manufacturer. Briefly, the plate was coated with capture antibody at 4 °C for 24 h. Next, the plate was blocked at room temperature (RT) for 1 h and incubated with sample culture supernatants at RT for 2 h. Then, the detection antibody was added, and the plate was incubated (2 h). The plate was washed 3 times, and avidin-HRP solution was added (20 min). Finally, TMB substrate solution was added to the plate and color development was stopped by a stop solution after 20 min. The signal was measured at 450 and 570 nm with a plate reader (BioTek Instruments, Winooski, VT, USA).

### 2.6. Western Blot Analysis

THP-1 macrophage cells in each well of 24 well plates were lysed with 100 µL of reducing lysis buffer (Laemmli reagent). The sample cell lysates were heated (95 °C) for 5 min. Then, proteins in the sample cell lysates were subject to sodium dodecyl sulphate polyacrylamide gel electrophoresis (SDS-PAGE) and transferred onto PVDF membrane (Thermo Fisher Scientific, Waltham, MA, USA). The membranes were then blocked with 5% bovine serum albumin diluted in tris-buffered saline containing 0.5% Tween 20 (BSA/TBST) at RT for 1 h, wash 3 times with washing buffer, and incubated with appropriate primary antibodies at 4 °C overnight. Primary antibodies included anti-IL-1β antibody (Cat No.) ab9722) (Abcam, Cambridge, UK), anti-ZIKV envelope protein (ZIKV E) antibody (Cat No. GTX133314) (GeneTex, Inc., Irvine, CA, USA), anti-ZIKV NS5 protein antibody (Cat No. GTX133312) (GeneTex, Inc., Irvine, CA, USA), anti-β-actin (Cat No. 3700) (Cell Signaling Technology, Danvers, MA, USA), anti-phospho-NF-κB (Cat No. 3033) (Cell Signaling Technology, Danvers, MA, USA), anti-NF-κB (Cat No. 8242) (Cell Signaling Technology, Danvers, MA, USA), anti-phospho-p38 MAPK (Cat No. 9216) (Cell Signaling Technology, Danvers, MA, USA), anti-p38 MAPK (Cat No. 8690) (Cell Signaling Technology, MA, USA), anti-phospho-p44/42 MAPK (ERK1/2) (Cat No. 4370) (Cell Signaling Technology, Danvers, MA, USA), anti-p44/42 MAPK (ERK1/2) (Cat No. 9107) (Cell Signaling Technology, Danvers, MA, USA), anti-phospho-SAPK/JNK (Cat No. 9255) (Cell Signaling Technology, Danvers, MA, USA), and anti-SAPK/JNK (Cat No. 9252) (Cell Signaling Technology, Danvers, MA, USA). Next, the membranes were probed with IRDye 800CW-conjugated anti-mouse IgG (Cat No. 926-32210) or IRDye 680RT-conjugated anti-rabbit IgG (Cat No. 926-68071) (LI–COR Biosciences, Lincoln, NE, USA) at RT for 2 h. Detection was performed by using the Odyssey^®^ CLx Imaging System (LI–COR Biosciences, Lincoln, NE, USA). The signal intensity of individual bands was quantitated by using the ImageJ software (version 1.51j8) (NIH, Bethesda, MD, USA) [30].

### 2.7. Immunofluorescence Study

THP-1 cells were seeded and differentiated on 12-mm diameter glass coverslips (Thermo Fisher Scientific, Braunschweig, Germany) placed in 24-well plates (3524, Corning, Kennebunk, ME, USA). After cell treatment, cell fixation was performed by removing the culture supernatants and adding 4% formaldehyde (diluted in phosphate buffer saline (PBS)) for 15 min at RT. The sample coverslips were washed 3 times with phosphate buffer saline (PBS) and the cells were then permeabilized with 0.3% TrironX-100 (diluted with PBS) at RT for 5 min. The sample coverslips were incubated at 4 °C for 24 h with appropriate primary antibodies (diluted in 1% BSA/TBST) at a dilution of 1: 500. Primary antibodies included anti-IL-1β antibody (Cat No. ab9722) (Abcam, Cambridge, UK), anti-ZIKV envelope protein (ZIKV E) antibody (Cat No. GTX133314) (GeneTex, Inc., Irvine, CA, USA), anti-phospho-NF-κB (Cat No. 3033) (Cell Signaling Technology, Danvers, MA, USA), anti-phospho-p38 MAPK (Cat No. 9216) (Cell Signaling Technology, Danvers, MA, USA), anti-p44/42 MAPK (ERK1/2) (Cat No. 9107) (Cell Signaling Technology, Danvers, MA, USA), anti-phospho-SAPK/JNK (Cat No. 9255) (Cell Signaling Technology, Danvers, MA, USA). The sample coverslips were then incubated with the mixture of specific secondary antibody (1:400), 1 μg/mL of 4′,6-diamidino-2-phenylindole, dihydrochloride (DAPI) (Cat No. 4083) (Cell Signaling Technology, Danvers, MA, USA) for nuclei staining, and with 1× of DyLight TM 594-Phalloidin (Cat No. 12877) (Cell Signaling Technology, Danvers, MA, USA) for F-actin staining for 2 h, at RT. Secondary antibodies included Alexa488-conjugated goat anti-rabbit IgG and Alexa488-conjugated goat anti-mouse IgG antibodies (Thermo Fisher Scientific, Waltham, MA, USA). Sample glass coverslips were mounted with anti-fade mounting media, and the signal was visualized by a fluorescent microscope (Axio Vert.A1) (Carl Zeiss AG, Oberkochen, Germany).

### 2.8. Plaque Assay

Vero cells grown in 6-well plates in DMEM complete media for 24 h were infected with culture supernatant containing ZIKV (10-fold dilution) at 37 °C for 2 h with constant agitation. Next, cells were overlaid with 1.2% methyl cellulose (MilliporeSigma, Burlington, MA, USA) containing 2% FBS-containing DMEM and incubated for 7 days. At day 7, the overlaid methyl cellulose was discarded, and cells were washed with PBS for one time. The cells were fixed with 3.7% formaldehyde (Thermo Fisher Scientific, Waltham, MA, USA) for 20 min at RT and stained with crystal violet (Thermo Fisher Scientific, Waltham, MA, USA) as previously described [29]. After washing, the plates were completely dried. Plaques were counted and calculated.

### 2.9. Statistical Analysis

All graphs from the raw data were created using The GraphPad Prism program (GraphPad Software Inc., San Diego, CA, USA). Statistical analysis was carried out by independent sample *t*-test. Data were presented as mean ± SD. * *p* < 0.05 indicated statistical significance between groups. Each experiment was performed at least 3 times independently.

## 3. Results

### 3.1. Effects of AP on the Viability of Human Macrophages

**Figure 1:**
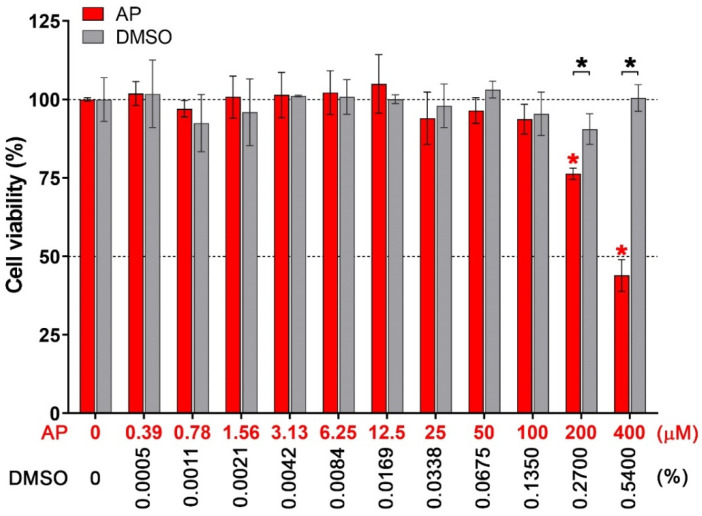
Effects of *AP* on THP-1 macrophage viability. TPA-activated THP-1 cells were treated with varied concentrations of AP (0–400 µM) for 48 h and subjected to MTT assay. DMSO (0–0.5400%) was used as a vehicle control. Data were obtained from three independent experiments. * *p* < 0.05 (red asterisk (compared with the untreated group), and black asterisk (compared with the DMSO-treated group)).

Since we aimed to examine the effect of AP on inhibiting ZIKV-induced IL-1β release in human macrophages, we initiated our investigation by testing the toxicity of the compound on THP-1 macrophages. Results from the MTT assay (Figure 1) demonstrated that AP at concentrations of 0.39 to 100 µM did not substantially affect the cell viability. The 50% cytotoxic concentration (CC50) value of AP for THP-1 macrophage was evaluated to be 279.53 µM. DMSO at all concentrations tested did not affect THP-1 macrophage viability. Although AP at 100, 50, and 25 µM did not exert significant cytotoxic effects on the cells, these concentrations showed a trend of reduced cell viability. Therefore, we chose completely non-toxic concentrations, which were 12.5, 6.25, 3.125, and 1.56 µM, for further experiments.

### 3.2. AP Reduces ZIKV-Induced Secretion of Interleukin 1β (IL-1β) from THP-1 Macrophages

**Figure 2:**
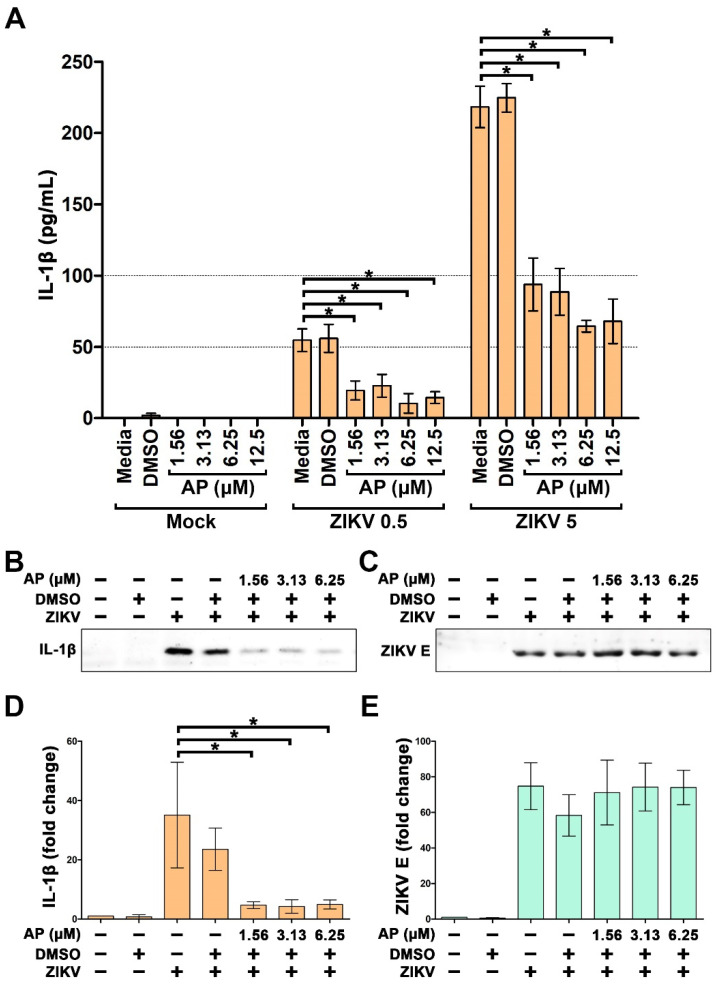
Inhibitory effects of AP on the secretion of IL-1β in the culture supernatants of TPA-activated THP-1 cells infected with ZIKV. (**A**) ELISA assay determining level of IL-1β in the culture supernatants collected from the cells infected with ZIKV at MOI 0.5 (ZIKV 0.5) or at MOI 5 (ZIKV 5) for 48 h. (**B**) Western blotting demonstrating immunoreactive bands of IL-1β in the supernatants of the cells infected with ZIKV at MOI 5 for 48 h. (**C**) Western blot analysis showing immunoreactive bands of ZIKV E protein in the culture supernatants of the cells infected with ZIKV at MOI 5 at for 48 h. (**D**) Quantitative analysis of immunoreactive bands of IL-1β in the culture supernatants of the cells infected with ZIKV at MOI 5 for 48 h. (**E**) Quantitative analysis of immunoreactive bands of ZIKV E protein in the culture supernatants of the cells infected with ZIKV at MOI 5 for 48 h. * *p* < 0.05 compared with the ZIKV-infected group (ZIKV-media). Densitometry scans and uncropped blots are presented in Supplementary Materials.

Since it has been reported that ZIKV infection can induce the immune cells to respond by secreting IL-1β into the extracellular environment leading to inflammation, we then tested the effect of AP on IL-1β secretion upon ZIKV infection. Results from ELISA assay showed that THP-1 macrophages cultured for 48 h exhibited undetectable level of IL-1β basal level in the culture supernatant and, thus, AP treatment caused no difference in IL-1β levels (Figure 2A, mock). When the cells were infected with ZIKV at MOI 0.5 (ZIKV 0.5) for 2 h followed by incubating with complete media for 48 h, the concentration of IL-1β was detected to be approximately 55 picogram per milliliter (pg/mL) which was equal to that of the culture supernatants collected from DMSO-treated cells (Figure 2A). However, when ZIKV-infected cells were exposed to AP at 1.56, 3.13, 6.25, and 12.5 µM for 48 h, the level of IL-1β was significantly decreased to 19.47 ± 6.67, 22.70 ± 7.97, 10.36 ± 6.82, and 14.40 ± 4.10 pg/mL, respectively (Figure 2A). The inhibitory effect of AP on IL-1β secretion could still be strong, even when THP-1 macrophages were infected with ZIKV at MOI 5 (ZIKV 5). Specifically, IL-1β in the culture supernatants collected from THP-1 macrophages infected with ZIKV 5 for 2 h followed by incubating with complete media for 48 h was detected to be greatly increased to 218.34 ± 14.51 pg/mL (Figure 2A). Cells treated with DMSO as a vehicle control for 48 h after infection generated 224.75 ± 10.01 pg/mL of IL-1β in the culture supernatants (Figure 2A). Interestingly, AP treatment exhibited strong inhibition of IL-1β, where AP at 1.56, 3.13, 6.35, and 12.5 µM could suppress IL-1β secretion to 93.78 ± 18.58, 88.64 ± 16.42, 64.51 ± 4.06, and 67.95 ± 15.57 pg/mL, respectively (Figure 2A). The maximal reduction (approximately 70.5% inhibition) of IL-1β was seen in the group treated with 6.25 µM of AP, and the inhibitory effect of AP seemed to be saturated since increasing the concentration of AP to 12.5 µM did not show any reduction trend. The inhibitory effect of AP was clearly observed to occur in a concentration-dependent manner when we further performed a 10-fold dilution to create the concentration range (0.000156–1.5625 μM) of AP (Figure S1). We further confirmed the ELISA results with a Western blot analysis to visualize the existence of IL-1β secreted in the culture supernatants. As expected, the immunoreactive band of IL-1β was detected in the culture supernatants of THP-1 macrophages with ZIKV infection, and AP at all concentrations effectively reduced the intensity of IL-1β-positive immunoreactive bands (Figure 2B). Densitometric quantification revealed that ZIKV at MOI 5 stimulated secretion of IL-1β in the culture supernatant by 35.07 ± 17.82-fold, but AP at 1.56, 3.13, and 6.35 µM strongly suppressed ZIKV-induced IL-1β secretion by 4.68 ± 1.13-, 4.23 ± 2.26-, and 4.92 ± 2.63-fold, respectively (Figure 2D). Western blot analysis also demonstrated that AP did not have any effect on viral production since ZIKV E protein in the culture supernatants was not different among all ZIKV-infected groups (Figure 2C,E).

**Figure 3:**
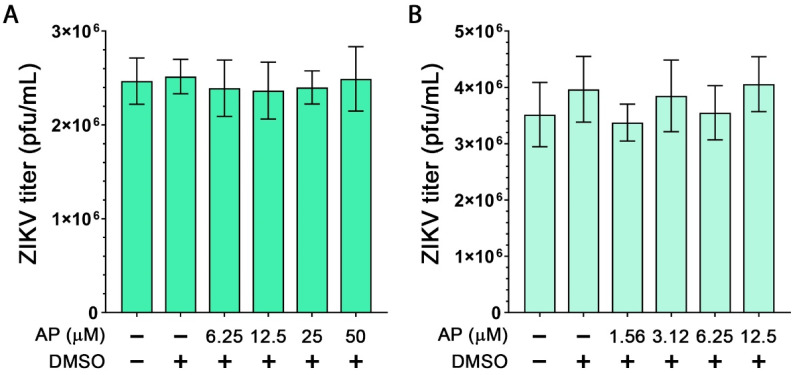
Effects of AP on ZIKV. (**A**) Virucidal activity of AP was determined by incubating ZIKV with complete media, DMSO, or various concentrations of AP at 37 °C for 2 h followed by plaque assay. (**B**) TPA-induced THP-1 cells were infected with ZIKV (MOI of 5) for 2 h. After infection, complete media, DMSO, or various concentrations of AP were added and incubated for 48 h. Sample supernatants were harvested to examine ZIKV viral productivity by plaque assay. Each condition was performed three times with duplicate plaque assay.

We further demonstrated that exposing ZIKV with different concentrations of AP for 2 h prior to plaque assay did not reduce ZIKV titer, indicating that AP is less likely to possess virucidal activity against ZIKV (Figure 3A). Consistent with this finding, treatment of AP following ZIKV infection for 48 h was demonstrated to have no significant difference in ZIKV titer (Figure 3B).

### 3.3. AP Reduces ZIKV-Induced Production of IL-1β in THP-1 Macrophages

**Figure 4:**
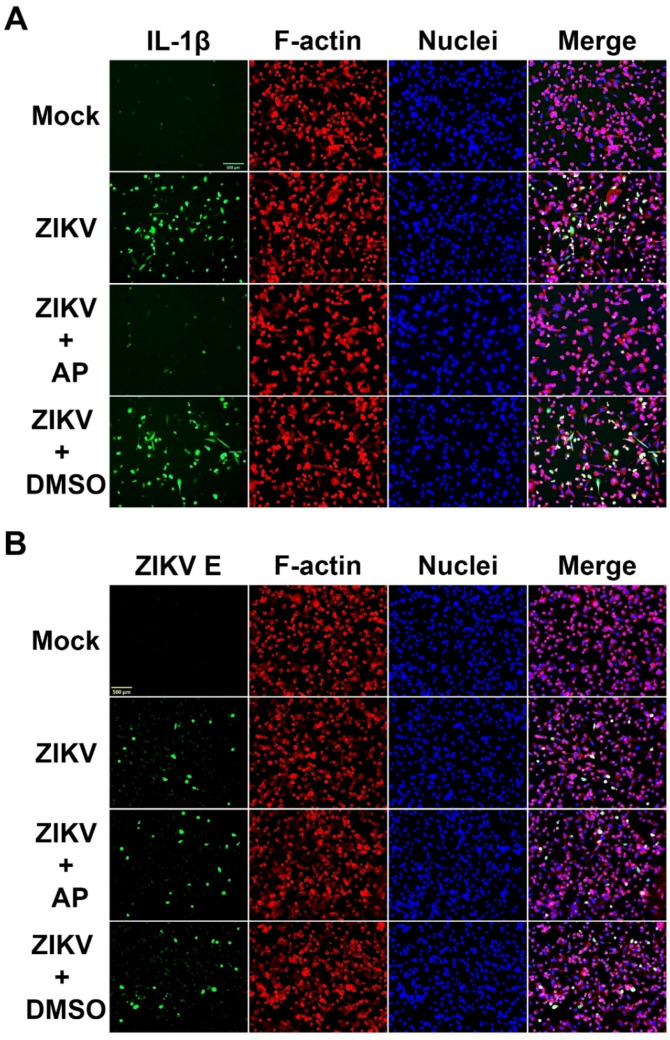
Immunofluorescence study examining the effects of AP on intracellular production of IL-1β (**A**) and ZIKV E protein (**B**) in ZIKV-infected THP-1 cells (5 MOI) for 48 h. Green is IL-1β or ZIKV E protein in the cells. Filamentous (F) actin (red) was stained with DyLight TM 594-Phalloidin, and nuclei of cells (blue) were stained with DAPI. Scale bar = 500 µm.

Based on data from ELISA assay and Western blot analysis revealing that AP treatment resulted in a drastic reduction in mature IL-1β secretion in the culture supernatants of ZIKV-infected THP-1, we hypothesized that AP may exert its inhibitory effects on the production of IL-1β. We therefore performed an immunofluorescence study to visualize the production of intracellular IL-1β in THP-1 macrophages infected with ZIKV at MOI 5. Results clearly showed that mock-infected cells showed very low positive basal level of intracellular IL-1β while ZIKV infection markedly increased the production of IL-1β in THP-1 macrophages (Figure 4A). The level of the cytokine in ZIKV-infected cells treated with DMSO was similar to that of ZIKV-infected cells without any treatment (Figure 4A). However, cells exposed to 6.25 µM of AP essentially inhibited ZIKV-induced production of IL-1β in THP-1 macrophages (Figure 4A). In contrast, the intracellular signal of ZIKV E protein was not different among ZIKV-infected cells, ZIKV-infected cells with AP treatment, and ZIKV-infected cells with DMSO control group (Figure 4B).

**Figure 5:**
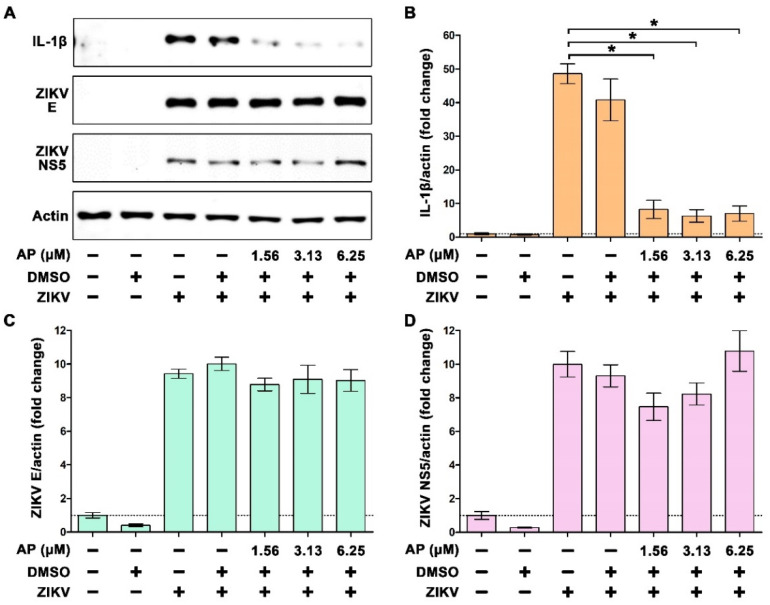
Western blot analysis examining the effects of AP on intracellular production of IL-1β, ZIKV E protein, and ZIKV NS5 protein in THP-1 macrophages infected with ZIKV at 5 MOI for 48 h. (**A**) Immunoreactive bands of IL-1β, ZIKV E protein, and ZIKV NS5 protein in the lysates of THP-1 macrophages infected with ZIKV at 5 MOI with or without post-treatment with AP at various concentrations (1.56, 3.13, and 6.25 µM). (**B**) Densitometric quantification of IL-1β immunoreactive bands. (**C**) Densitometric quantification of ZIKV E protein immunoreactive bands. (**D**) Densitometric quantification of ZIKV NS5 protein immunoreactive bands. Actin was used as an internal control and for normalization. * *p* < 0.05 compared to ZIKV-infected group. Densitometry scans and uncropped blots are presented in Supplementary Materials.

These results from the immunofluorescence study were consistent with those from the Western blot analysis which revealed that ZIKV infection strongly induced the production of IL-1β in the cells, and AP at all selected concentrations strongly suppressed this event (Figure 5A). We noticed that even the lowest concentration of AP (1.56 µM) could effectively inhibit intracellular production of IL-1β in response to ZIKV infection (Figure 5B). In line with the results from the immunofluorescence study, Western blot analysis verified that AP treatment showed no significant effect on ZIKV production in the cells, since there was no change in intracellular ZIKV E protein production (Figure 5C) and ZIKV NS5 protein production (Figure 5D).

### 3.4. Inhibitory Effects of AP on ZIKV-Induced IL-1β Is Not Regulated through the Inhibition of NF-κB Signaling

**Figure 6:**
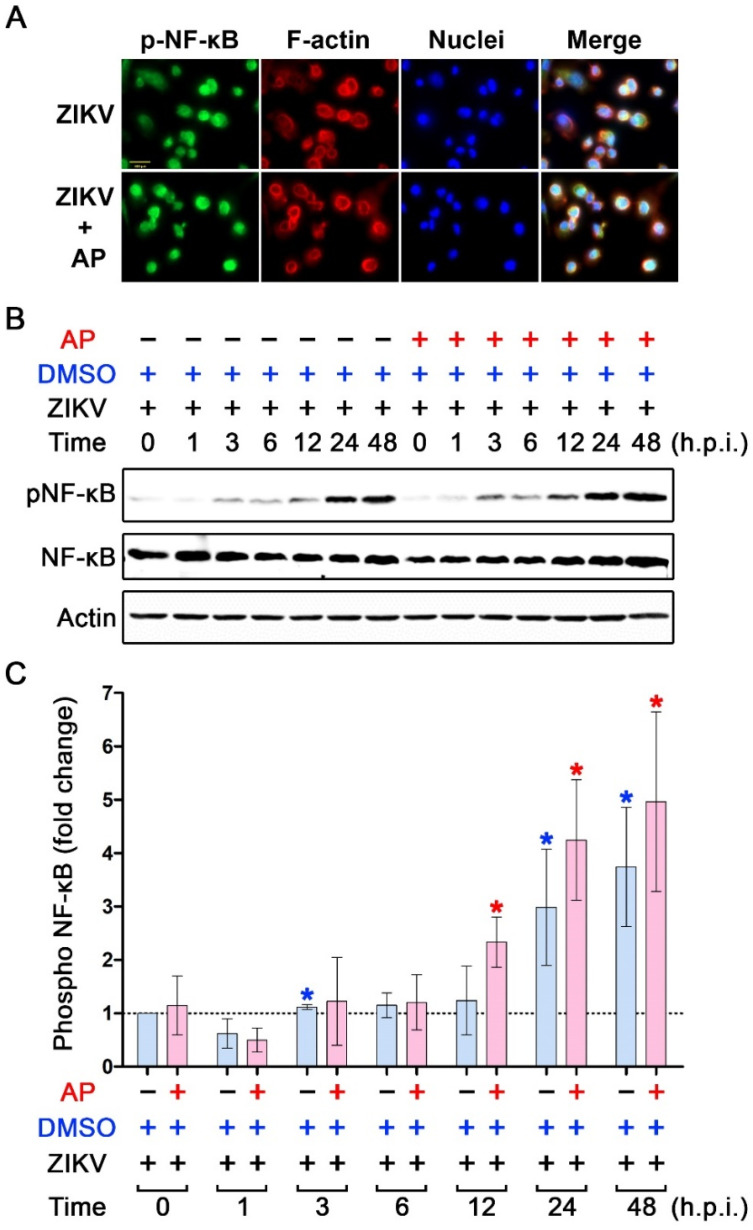
Effects of AP on the activation status of NF-κB in THP-1 macrophages infected with ZIKV. (**A**) Immunofluorescence study detecting the presence of NF-κB (green) in the nucleus of THP-1 macrophages infected with ZIKV at MOI 5 for 48 h. F-actin (red) was stained with DyLight TM 594-Phalloidin, and nuclei of cells (blue) were stained with DAPI. Scale bar = 500 µm. (**B**) Western blot analysis detecting phosphorylated NF-κB (p-NF-κB), total NF-κB (NF-κB), and actin in the lysates of THP-1 macrophages infected with ZIKV at 5 MOI with or without post-treatment with 6.25 µM AP at various time points (0–48 h). (**C**) Densitometric quantification of p-NF-κB immunoreactive bands versus total NF-κB in the lysates of THP-1 macrophages infected with ZIKV at 5 MOI with or without post-treatment with 6.25 µM of AP at various time points (0–48 h). Actin was used as an internal control. * *p* < 0.05 (red asterisk (compared to the AP-treated group at 0 h) and blue asterisk (compared to the DMSO control group at 0 h)). h.p.i. = hour(s) post-infection. Densitometry scans and uncropped blots are presented in Supplementary Materials.

Since ZIKV infection has been reported to stimulate IL-1β production, in part through activation of NF-κB signaling, we thus tested whether AP can suppress ZIKV-induced activation of NF-κB. We first performed an immunofluorescence study to monitor the phosphorylation status of NF-κB and its translocation from the cytosol to the nucleus upon infection with ZIKV. We found that 48 h post-infection with ZIKV, the signal of phosphorylated NF-κB (p-NF-κB) was detected in the nuclei of THP-1 macrophages (Figure 6A). Surprisingly, the presence of AP did not create any different intensity of nuclear p-NF-κB compared to that of ZIKV-infected cells without any treatment (Figure 6A). Additionally, we performed Western blotting to monitor the phosphorylation status at various time points from the beginning of ZIKV infection to 48 h post-infection. Data confirmed that phosphorylation of NF-κB was gradually increased over time after ZIKV infection, and the phosphorylation of this protein reached a maximal level (approximately 5-fold) at 48 h post-infection without significantly affecting the expression level of total NF-κB (Figure 6B,C). Importantly, it was verified that AP had no inhibitory activity against ZIKV-induced NF-κB phosphorylation in THP-1 macrophages (Figure 6B,C). TPA-induced THP-1 macrophages without Zika virus infection showed no obvious NF-κB phosphorylation over 48 h. DMSO and *AP* treatment did not demonstrate any reduction trend of this low basal level of NF-κB phosphorylation (Figure S2).

### 3.5. AP Selectively Suppresses ZIKV-Induced Activation of p38 MAPK

**Figure 7:**
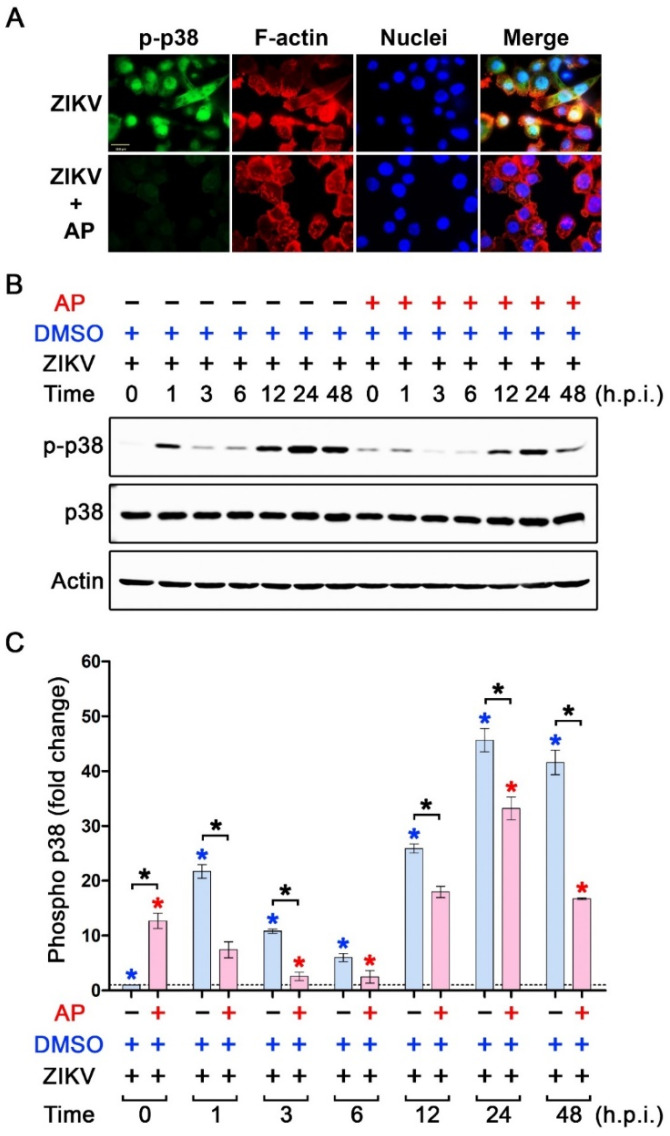
Effects of AP on the activation status of p38 kinase in ZIKV-infected THP-1 macrophages. (**A**) Immunofluorescence study detecting the presence of phosphorylated p38 kinase (p-p38) (green) in the nucleus of ZIKV-infected THP-1 macrophages (MOI 5) for 1 h. F-actin (red) was stained with DyLight TM 594-Phalloidin, and nuclei of cells (blue) were stained with DAPI. Scale bar = 500 µm. (**B**) Western blot analysis detecting p-p38 kinase, total p38 kinase (p38), and actin in the lysates of THP-1 macrophages infected with ZIKV at 5 MOI with or without post-treatment with 6.25 µM of AP at various time points (0–48 h). (**C**) Densitometric quantification of p-p38 immunoreactive bands versus total p38 in the lysates of THP-1 macrophages infected with ZIKV at 5 MOI with or without post-treatment with 6.25 µM of AP at various time points (0–48 h). Actin was used as an internal control. * *p* < 0.05 (red asterisk (compared to the AP-treated group at 0 h), blue asterisk (compared to the DMSO control group at 0 h), and black asterisk (compared between AP-treated group and DMSO control group at each time point)). h.p.i. = hour(s) post-infection. Densitometry scans and uncropped blots are presented in Supplementary Materials.

**Figure 8:**
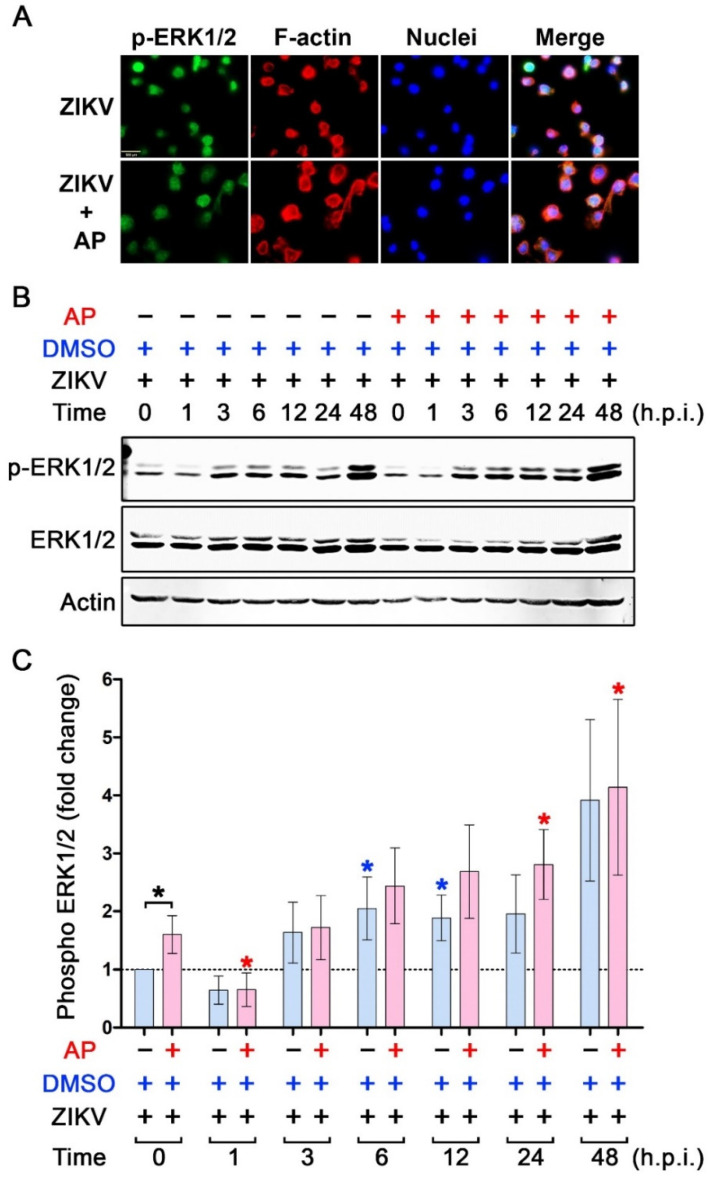
Effects of AP on the activation status of pERK1/2 kinase in THP-1 macrophages infected with ZIKV. (**A**) Immunofluorescence study detecting the presence of phosphorylated ERK1/2 kinase (p-ERK1/2) (green) in the nucleus of THP-1 macrophages infected with ZIKV at MOI 5 for 24 h. F-actin (red) was stained with DyLight TM 594-Phalloidin, and nuclei of cells (blue) were stained with DAPI. Scale bar = 500 µm. (**B**) Western blot analysis detecting p-ERK1/2 kinase, total ERK1/2 kinase (ERK1/2), and actin in the lysates of THP-1 macrophages infected with ZIKV at 5 MOI with or without post-treatment with 6.25 µM of AP at various time points (0–48 h). (**C**) Densitometric quantification of p-ERK1/2 immunoreactive bands versus total ERK1/2 in the lysates of THP-1 macrophages infected with ZIKV at 5 MOI with or without post-treatment with 6.25 µM of AP at various time points (0–48 h). Actin was used as an internal control. * *p* < 0.05 (red asterisk (compared to the AP-treated group at 0 h), blue asterisk (compared to the DMSO-treated group at 0 h), and black asterisk (compared between AP-treated group and DMSO control group at each time point)). h.p.i. = hour(s) post-infection. Densitometry scans and uncropped blots are presented in Supplementary Materials.

**Figure 9:**
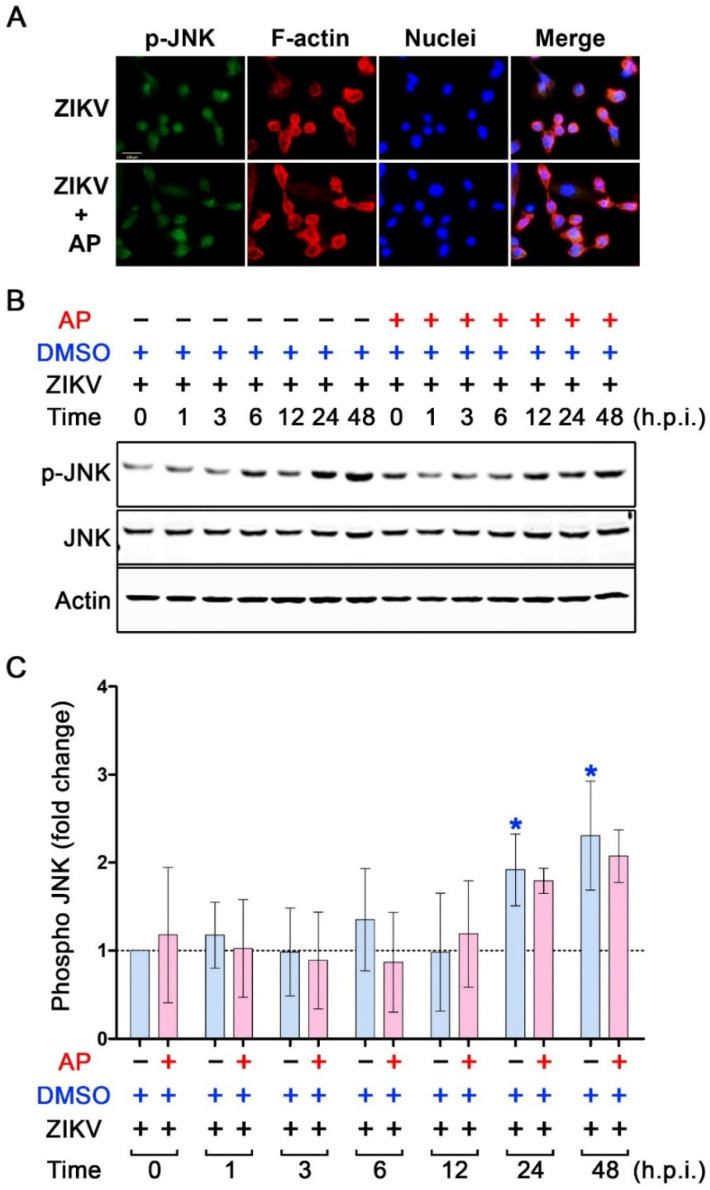
Effects of AP on the activation status of JNK kinase in THP-1 macrophages infected with ZIKV. (**A**) Immunofluorescence study detecting the presence of phosphorylated JNK kinase (p-JNK) (green) in the nucleus of THP-1 macrophages infected with ZIKV at MOI 5 for 24 h. F-actin (red) was stained with DyLight TM 594-Phalloidin, and nuclei of cells (blue) were stained with DAPI. Scale bar = 500 µm. (**B**) Western blot analysis detecting p-JNK kinase, total JNK kinase (JNK), and actin in the lysates of THP-1 macrophages infected with ZIKV at 5 MOI with or without post-treatment with 6.25 µM of AP at various time points (0–48 h). (**C**) Densitometric quantification of p-JNK immunoreactive bands versus total JNK in the lysates of THP-1 macrophages infected with ZIKV at 5 MOI with or without post-treatment with 6.25 µM of AP at various time points (0–48 h). Actin was used as an internal control. * *p* < 0.05 (blue asterisk (compared to the DMSO-treated group at 0 h)). h.p.i. = hour(s) post-infection. Densitometry scans and uncropped blots are presented in Supplementary Materials.

We further determined the effect of AP on the activation status of MAP kinases in ZIKV-infected THP-1 macrophages. Data from an immunofluorescence study disclosed that ZIKV infection rapidly activated p38 phosphorylation after 1 h of infection, but this ZIKV-induced p38 phosphorylation could barely be detected in ZIKV-infected cells with the presence of AP (Figure 7A). We confirmed these results with a Western blot analysis where we monitored the phosphorylation status of p38 in THP-1 macrophages over the course of 48 h after ZIKV infection with or without the presence of AP. We observed that exposing THP-1 macrophages to ZIKV rapidly induced p38 phosphorylation after 1 h by approximately 20-fold compared to the base line at 0 h (Figure 7B,C). ZIKV-infected THP-1 macrophages exhibited dynamic changes in p38 phosphorylation status. Specifically, at 3 h and 6 h post-infection, p38 phosphorylation decreased dramatically before returning to be around 25-fold at 12 h post-infection (Figure 7B,C). Phosphorylation of p38 peaked at 24 h (about 45-fold compared to the base line at 0 h) and tended to slightly decline to approximately 40-fold (Figure 7B,C). However, AP treatment could significantly reduce ZIKV-induced p38 phosphorylation at almost all time points (1 h, 3 h, 12 h, 24 h, and 48 h post-infection) (Figure 7B,C). Interestingly, AP exhibited no significant effect on ZIKV-induced phosphorylation (at all time points post-infection) of ERK1/2 MAP kinase (Figure 8) and JNK MAP kinase (Figure 9). Like NF-κB, we did not observe an increase in phosphorylation of p38, ERK1/2, and JNK over the course of 48 h in TPA-induced THP-1 macrophage without Zika virus infection, and the basal level patterns of phosphorylated kinases were similar in DMSO and AP-treated groups (Figure S2).

## 4. Discussion

Although ZIKV infection that causes no symptom in humans is considered a mild and self-limiting febrile disease, accumulated cases of unexpectedly severe clinical complications have made this causative agent a cause of global public health concern. ZIKV infection-related complications include Guillain–Barré syndrome (an adult neurological autoimmune disease) and microcephaly in infants [31,32,33]. Moreover, ZIKV infection can lead to chorioretinal atrophy and optic nerve abnormalities in infants [34,35]. ZIKV infects many different cells in the body and induces the release of cytokines, especially interleukin 1β (IL-1β), which contribute to tissue and organ abnormalities [10,36,37,38,39]. Specifically, ZIKV-infected cells were shown to secrete high level of IL-1α and IL-1β, which might induce cell death [12,40]. In addition, serum IL-1β was found in nonpregnant patients with ZIKV infection [14,41]. Undoubtedly, prevention approaches and treatment strategies are being developed.

In addition to controlling exposure to mosquito vectors [42,43], there have been extensive studies attempting to develop vaccines, antiviral agents, and antibody therapies to combat ZIKV infection [6,41,44]. However, research aspects related to development of treatments aiming to mitigate the inflammatory effects of ZIKV-induced IL-1 have not gained much attention. Previous animal studies have provided convincing data that treating pregnant animals with IL-1 receptor antagonists could effectively defend against placental and neurodevelopmental abnormalities caused by a high level of inflammation in the uterus [45,46,47]. For ZIKV infection, IL-1 receptor antagonist therapy was examined to alleviate dysfunction of the placenta and perinatal injury caused by ZIKV infection [23]. These studies suggest that IL-1 production induced by ZIKV infection plays a significant role in causing organ damages and should be a treatment target for reducing adverse outcomes.

On the basis that AP possesses strong anti-inflammatory activities in several different models [25,26,27], we thus explored the effects of this flavonoid on suppressing the release of IL-1β from TPA-activated THP-1 macrophages, in response to ZIKV infection. We found that the toxic concentration range of AP was very high, indicating that AP is relatively non-toxic to THP-1 macrophages. Consistent with previous reports, results from ELISA assays demonstrated that IL-1β was considerably elevated in the supernatant when the cells were infected with ZIKV. The level of IL-1β was greatly increased when the cells were infected with higher multiplicity of infections of ZIKV. The level of IL-1β in the supernatant of ZIKV-infected THP-1 macrophages was tested by Western blotting confirming that ZIKV infection can activate human macrophage response by secreting IL-1β into the extracellular environment. Interestingly, the level of IL-1β in the culture supernatant was dramatically low in ZIKV-infected THP-1 cells exposed to (post-treatment) AP at relatively low concentrations. These data raised the question whether AP reduces ZIKV production, resulting in the observed decrease in IL-1β in the culture supernatants. However, the level of ZIKV E protein in the culture supernatants of the ZIKV-infected THP-1 macrophages was approximately equal in all groups. Moreover, data from plaque assay clearly indicated that AP did not decrease the production of infectious virions. Therefore, it is evident that the action of AP on lowering ZIKV-induced IL-1β in the culture supernatants of THP-1 macrophages is not caused by inhibition of ZIKV production. One possible mechanism may be from the conserved ability of AP in inhibiting the activation of NLRP3 inflammasome leading to the suppression of the cleavage of pro-IL-1β by active caspase 1, as reported in a different inflammatory model [26]. Alternatively, AP may act at the earlier steps to repress the production of IL-1β. To prove our hypotheses, we first sought to determine the changes in intracellular expression of IL-1β. Results from an immunofluorescence study illustrated that ZIKV infection activated intracellular production of IL-1β, and this ZIKV-induced IL-1β production was almost completely reduced in ZIKV-infected THP-1 macrophages with AP treatment. We established that AP had no virucidal activity. Likewise, AP had no effect on ZIKV production inside the infected cells, since the intracellular signal of ZIKV E protein was not different among ZIKV-infected cells (with or without the presence of AP), suggesting that AP inhibits ZIKV-induced IL-1β production by other means. Detection of IL-1β in the cell lysates by Western blot analysis clearly supported that AP treatment effectively inhibited the production of IL-1β without affecting the level of ZIKV E protein and NS5 protein, verifying that the inhibitory effects of AP on IL-1β is not caused by inhibition of ZIKV replication. Additionally, considering the role of ZIKV NS5 protein in directly interacting and activating NLRP3 inflammasome leading to IL-1β activation, our data showing that AP did not reduce the intracellular level of ZIKV NS5 protein further explains that suppression of IL-1β activation by AP is not due to downregulation of ZIKV NS5 protein. Our findings are consistent with a previous study where it was shown that AP attenuated the synthesis of IL-1β at the mRNA and protein levels in LPS-primed TPA-differentiated THP-1 cells [26].

As a step towards defining the mechanism of action of AP at the molecular level, we first focused on the NF-κB signaling pathway, which is a major pathway responsible for stimulating the production of IL-1β in response to pathogen exposure [48,49,50]. It is possible that AP may hamper this signaling to block IL-1β production. We found that ZIKV infection induced activation and nuclear translocation of NF-κB. ZIKV infection increasingly activated NF-κB phosphorylation over time; it was remarkably high at 24 h and 48 h post-infection. Surprisingly, AP treatment did not inhibit ZIKV-induced NF-κB phosphorylation at all time points, indicating that the inhibitory effects of AP on ZIKV-induced IL-1β production is less likely associated with direct inhibition of NF-κB activation.

Previous studies have demonstrated that ZIKV infection or the virus’ NS5 protein triggered intracellular production of ROS which further induced inflammasome activation, which is responsible for IL-1β production [20]. Notably, p38 MAPK is preferentially activated by extracellular stresses and, thus, elevation of intracellular ROS can actively stimulate p38 MAPK to play roles in stress responses [51,52]. Therefore, we determined whether AP has inhibitory effects on p38 MAPK activation by monitoring p38 MAPK phosphorylation status upon ZIKV infection. As expected, AP treatment could significantly inhibit p38 phosphorylation induced by ZIKV infection without exhibiting any effect on the ZIKV-induced phosphorylation of ERK1/2 and JNK MAP kinases. Our data were consistent with previous reports showing that p38 MAPK is markedly induced to stimulate inflammatory response, including secretion of IL-1β in retinal Müller cells infected with ZIKV, and inhibition of ZIKV-induced p38 MAPK effectively blocked inflammation [53]. Similar results were seen in dengue virus (DENV) infection, where administration of SB203580, a p38 MAPK inhibitor, strongly limited inflammation, prevented an increase in hematocrit, lymphopenia, and liver damage, and improved the survival rate of DENV-infected mice [54,55]. An additional study demonstrated that p38 MAPK inhibition effectively reduced inflammasome and caspase-1 activation induced by ribotoxic stress [56]. Specifically for IL-1β, it has been proved that activation of p38 MAPK plays a role in inducing IL-1β transcription through activating C/EBP/NFIL-6 transcription factors in LPS-induced monocytic cell lines; thus, inhibiting intracellular activation of this signaling pathway could effectively suppress transcription of IL-1β in a dose-dependent manner [57]. These studies support our current observations that AP may suppress the ZIKV-induced synthesis of IL-1β, at least in part, through inhibiting the activation of intracellular p38 MAPK. Taken together, our study provides promising information suggesting that AP is an interesting plant-derived compound to be developed as a novel therapy for mitigating ZIKV-induced IL-1β production and IL-1β-related pathological abnormalities.

## Data Availability

The data presented in this study are available in this article.

## Supplementary Materials

The following supporting information can be downloaded at: https://www.mdpi.com/article/10.3390/pharmaceutics14122800/s1. Table S1: Structure and mass of Alpinetin (7-Hydroxy-5-methoxyflavanone). Figure S1: ELISA assay determining level of IL-1β in the culture supernatants collected from THP-1 macrophages infected with ZIKV at MOI 5 (ZIKV 5) with the presence of various concentrations of AP (0 to 1.5625 μM) for 48 h. Figure S2: Determination of effects of AP on the phosphorylation status of NF-κB, p38, ERK1/2, and JNK in TPA-induced THP-1 macrophage over the course of 48 h without Zika infection.
