# Supplementary material for: Alpinetin Suppresses Zika Virus-Induced Interleukin-1β Production and Secretion in Human Macrophages
Source: Pharmaceutics. 2022 Dec 14;14(12):2800. doi: 10.3390/pharmaceutics14122800 (PMC9782830; doi:10.3390/pharmaceutics14122800)

Supplementary data

Table S1. Structure and mass of Alpinetin (7-Hydroxy-5-methoxyflavanone).

Compound	Structures	Molecular weight (MW)
Alpinetin (7-Hydroxy-5-methoxyflavanone) <br>Purity (HPLC-DAD, 300nm) = 99.3%	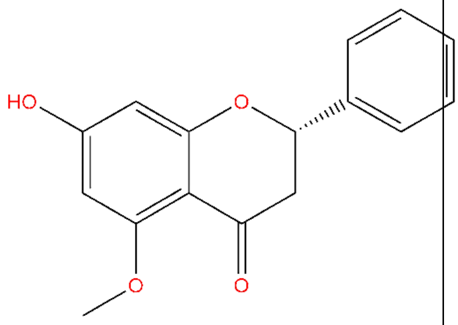	270.284 g/mol

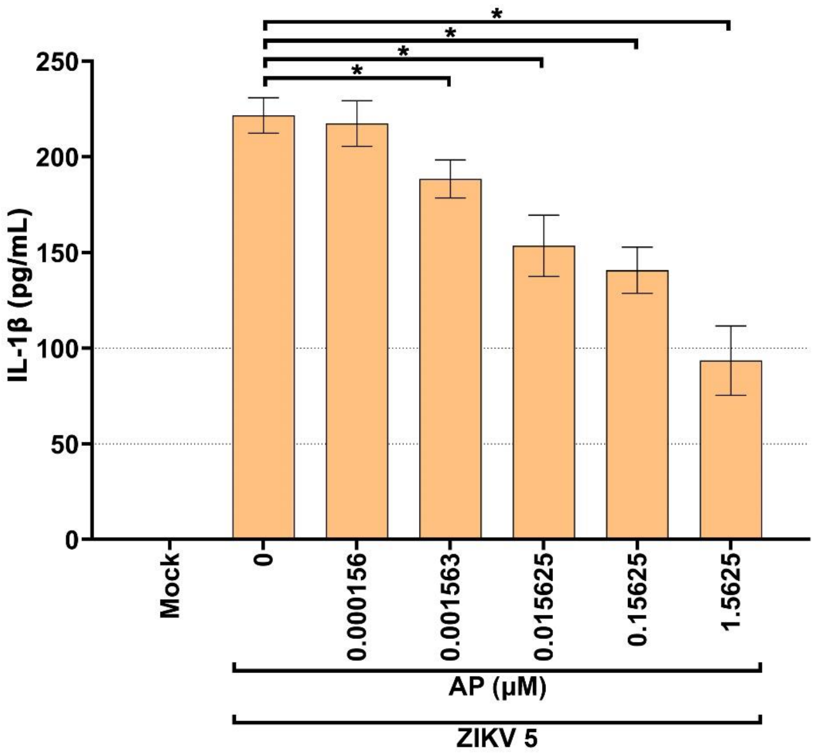

Figure S1. ELISA assay determining level of IL-1β in the culture supernatants collected from THP-1 macrophages infected with ZIKV at MOI 5 (ZIKV 5) with the presence of various concentrations of AP (0 to 1.5625 μM) for 48 h.

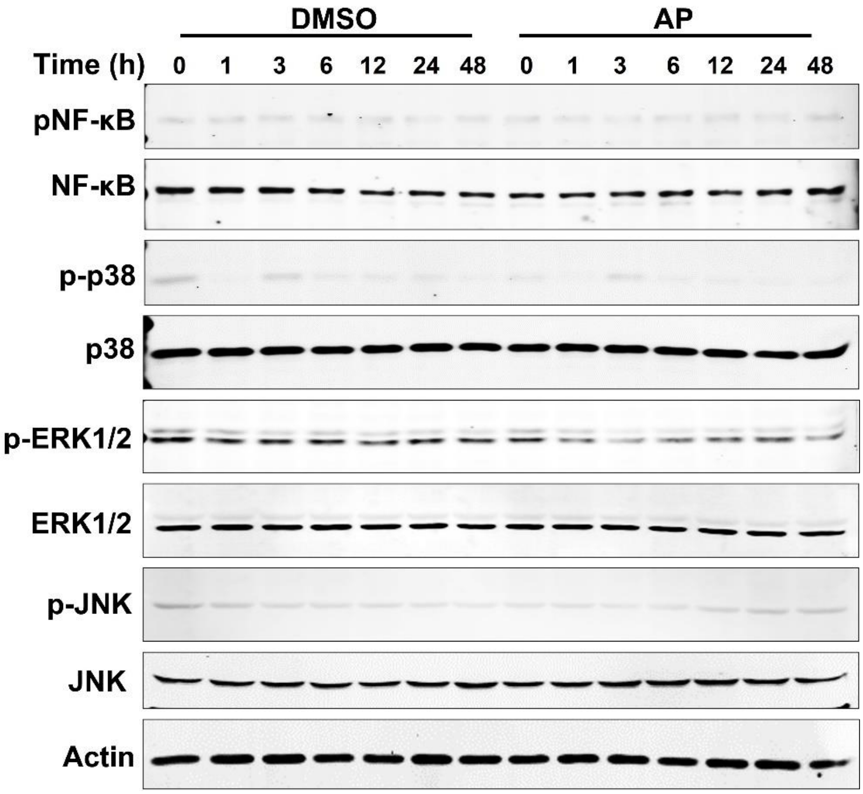

Figure S2. Determination of effects of AP on the phosphorylation status of NF-κB, p38, ERK1/2, and JNK in TPA-induced THP-1 macrophage over the course of 48 h without Zika infection. Western blot analysis detecting phosphorylated NF-κB (pNF-κB), total NF-κB (NF-κB), phosphorylated p38 (p-p38), total p38 (p38), phosphorylated ERK1/2 (p-ERK1/2), total ERK1/2 (ERK1/2), phosphorylated JNK (p-JNK), total JNK (JNK), and actin in the lysates of TPA-induced THP-1 macrophages treated with 6.25 μM AP at various time points (0-48 h).

Original Western blot for Figure 2B

Immunoblot: IL-1β

Red box represents the cropped image used in manuscript figure 2B.

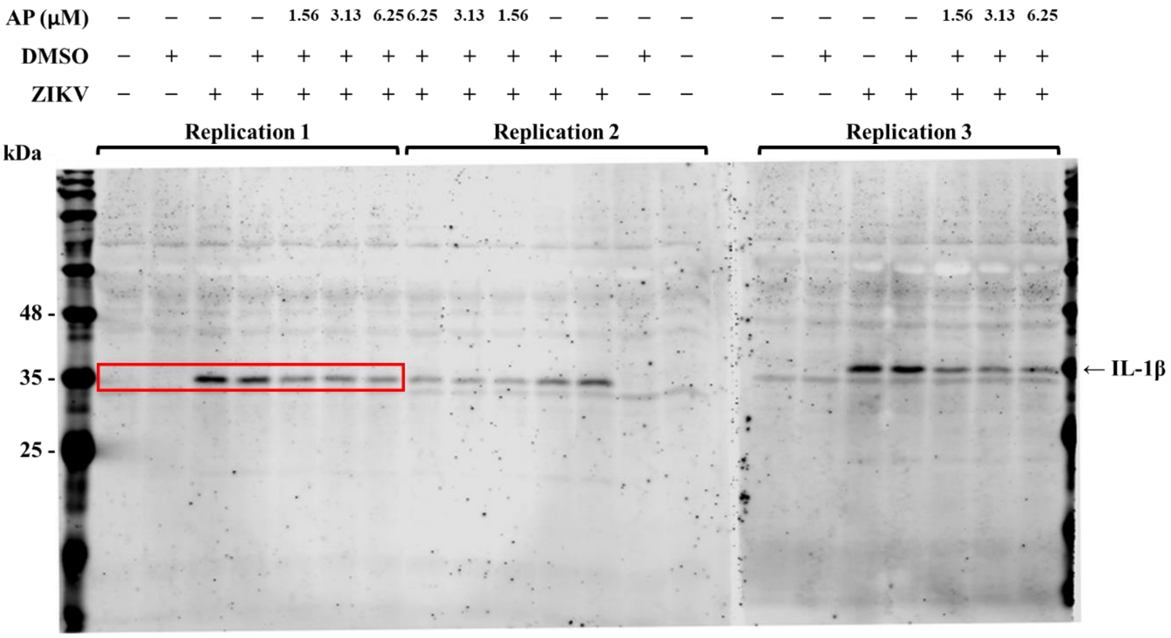

Ratio of Western blot for Figure 2D: IL-1β (fold change)

Mock						ZIKV 5														
Media			DMSO			Media			DMSO			AP (μM)								
												1.56			3.13			6.25		
1.00	1.00	1.00	0.57	1.53	0.11	20.77	55.04	29.39	15.67	25.18	29.72	3.53	5.79	4.73	3.49	6.76	2.43	2.24	7.50	5.00

Original Western blot for Figure 2C

Immunoblot: ZIKV E

Red box represents the cropped image used in manuscript figure 2C.

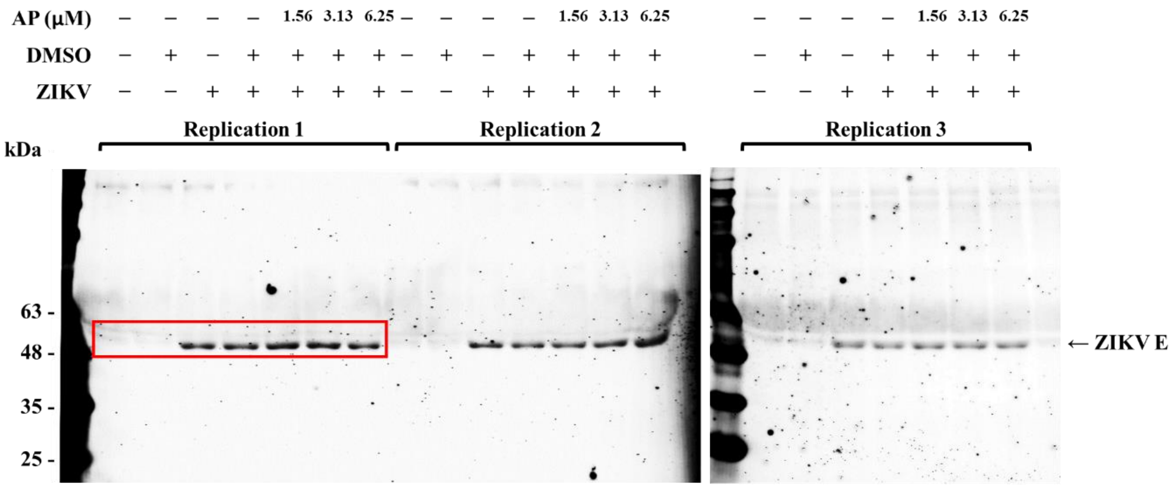

Ratio of Western blot for Figure 2E: ZIKV E (fold change)

Mock						ZIKV 5														
Media			DMSO			Media			DMSO			AP (μM)								
												1.56			3.13			6.25		
1.00	1.00	1.00	0.27	0.97	0.46	78.35	50.28	95.43	77.45	37.33	60.15	103.74	40.76	68.88	99.83	54.29	68.61	74.25	90.64	57.17

Original Western blot for Figure 5A

Immunoblot: IL-1β

Red box represents the cropped image used in manuscript figure 5A: IL-1β.

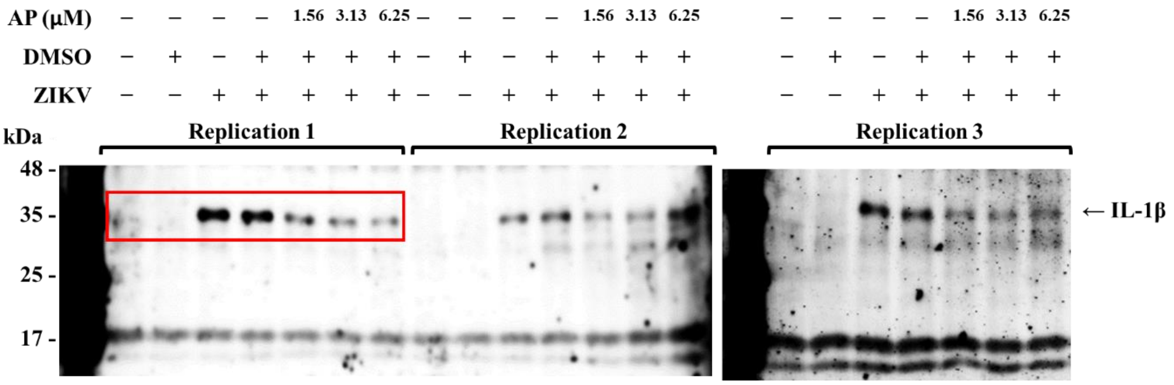

Original Western blot for Figure 5A

Immunoblot: ZIKV E

Red box represents the cropped image used in manuscript figure 5A: ZIKV E.

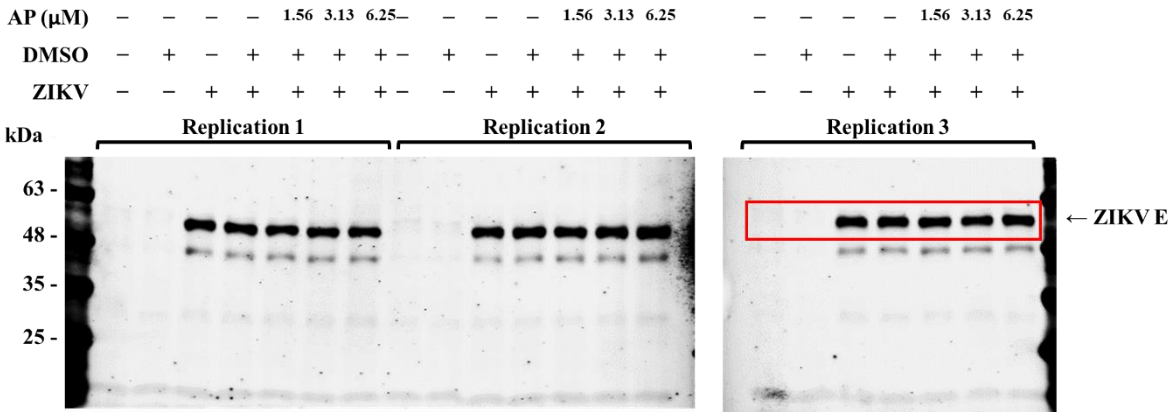

Original Western blot for Figure 5A

Immunoblot: ZIKV NS5

Red box represents the cropped image used in manuscript figure 5A: ZIKV NS5.

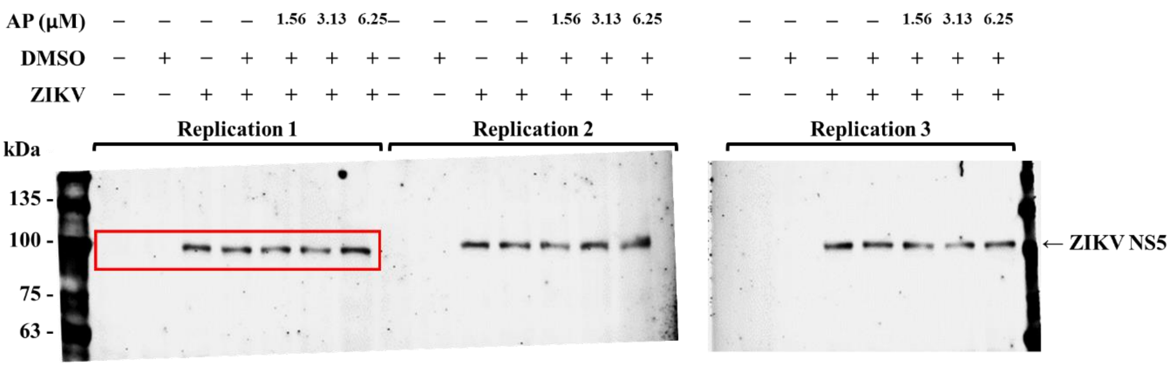

Original Western blot for Figure 5A

Immunoblot: Actin

Red box represents the cropped image used in manuscript figure 5A: Actin.

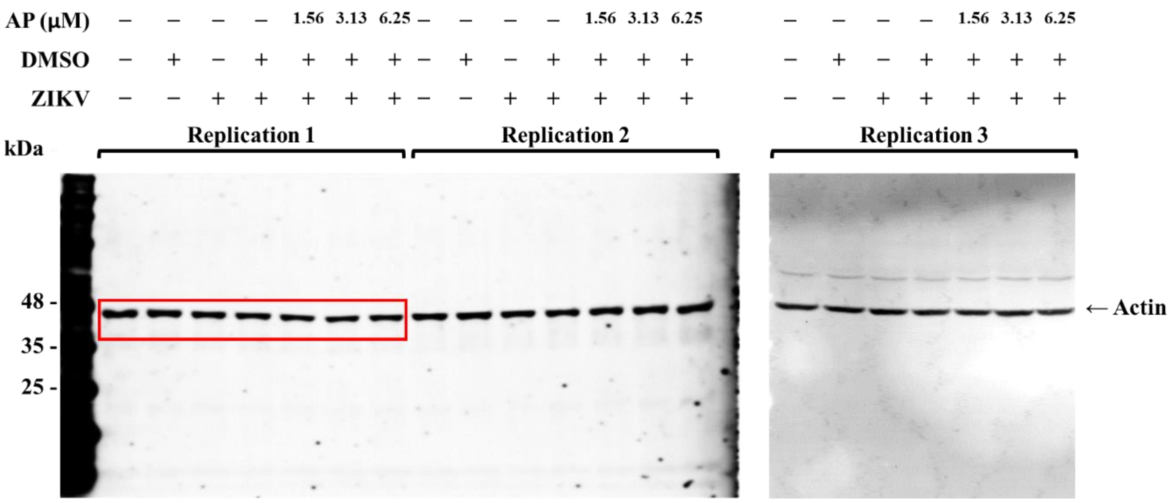

Ratio of Western blot for Figure 5B: IL-1β/actin (fold change)

Mock						ZIKV 5														
Media			DMSO			Media			DMSO			AP (μM)								
												1.56			3.13			6.25		
0.61	1.52	0.87	0.96	0.97	0.37	53.52	48.9	43.35	48.92	28.53	44.96	9.04	3.15	12.5	9.8	3.43	5.62	4.55	4.97	11.47

Ratio of Western blot for Figure 5C: ZIKV E/actin (fold change)

Mock						ZIKV 5														
Media			DMSO			Media			DMSO			AP (μM)								
												1.56			3.13			6.25		
0.78	0.91	1.31	0.25	0.52	0.44	9.69	9.7	8.88	10.28	10.53	9.22	9.52	8.26	8.56	10.77	8.31	8.2	10.13	7.91	9.03

Ratio of Western blot for Figure 5D: ZIKV NS5/actin (fold change)

Mock						ZIKV 5														
Media			DMSO			Media			DMSO			AP (μM)								
												1.56			3.13			6.25		
0.54	1.19	1.27	0.31	0.27	0.25	9.28	9.2	11.5	8.93	8.4	10.6	8.48	5.85	8.06	9.38	8.21	7.1	13.15	9.23	9.96

Original Western blot for Figure 6B

Immunoblot: pNF-κB

Red box represents the cropped image used in manuscript figure 6B: pNF-κB.

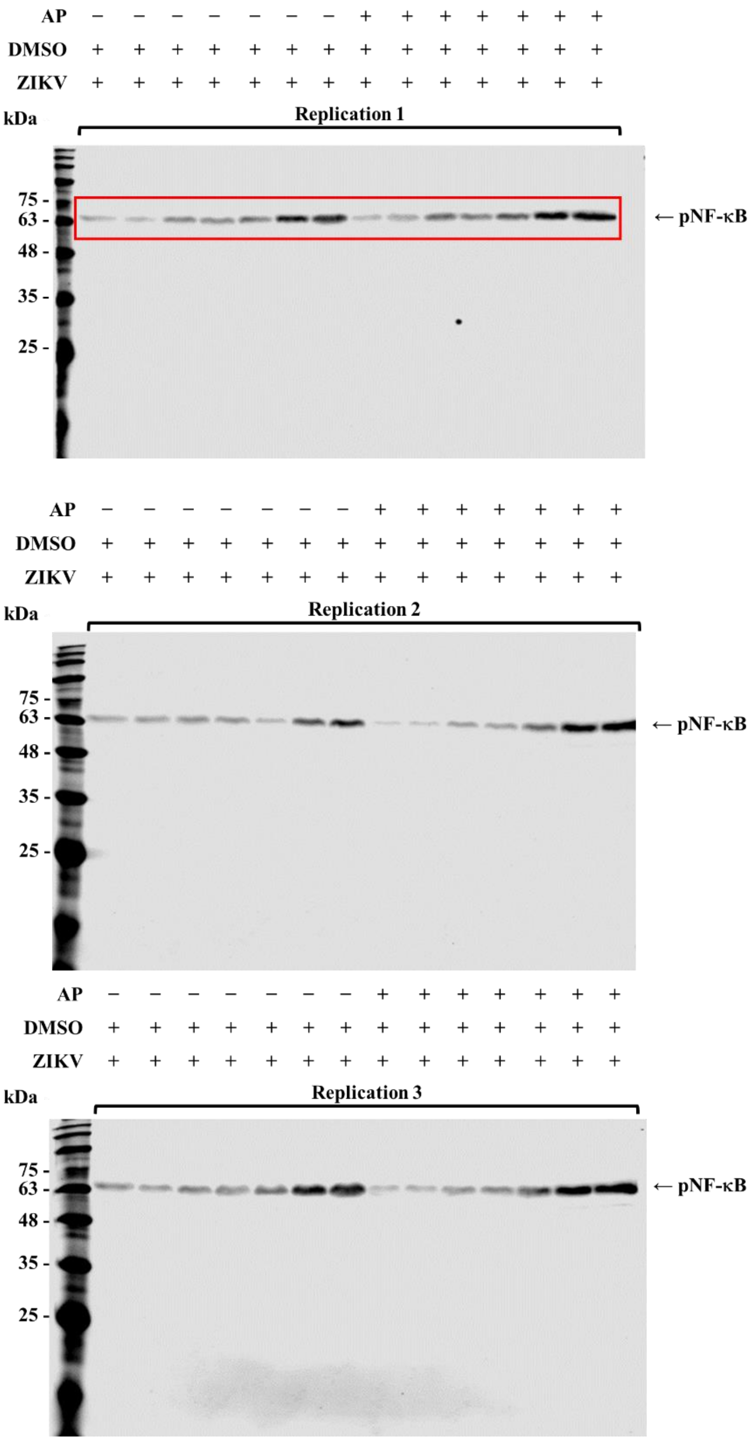

Original Western blot for Figure 6B

Immunoblot: NF-κB

Red box represents the cropped image used in manuscript figure 6B: NF-κB.

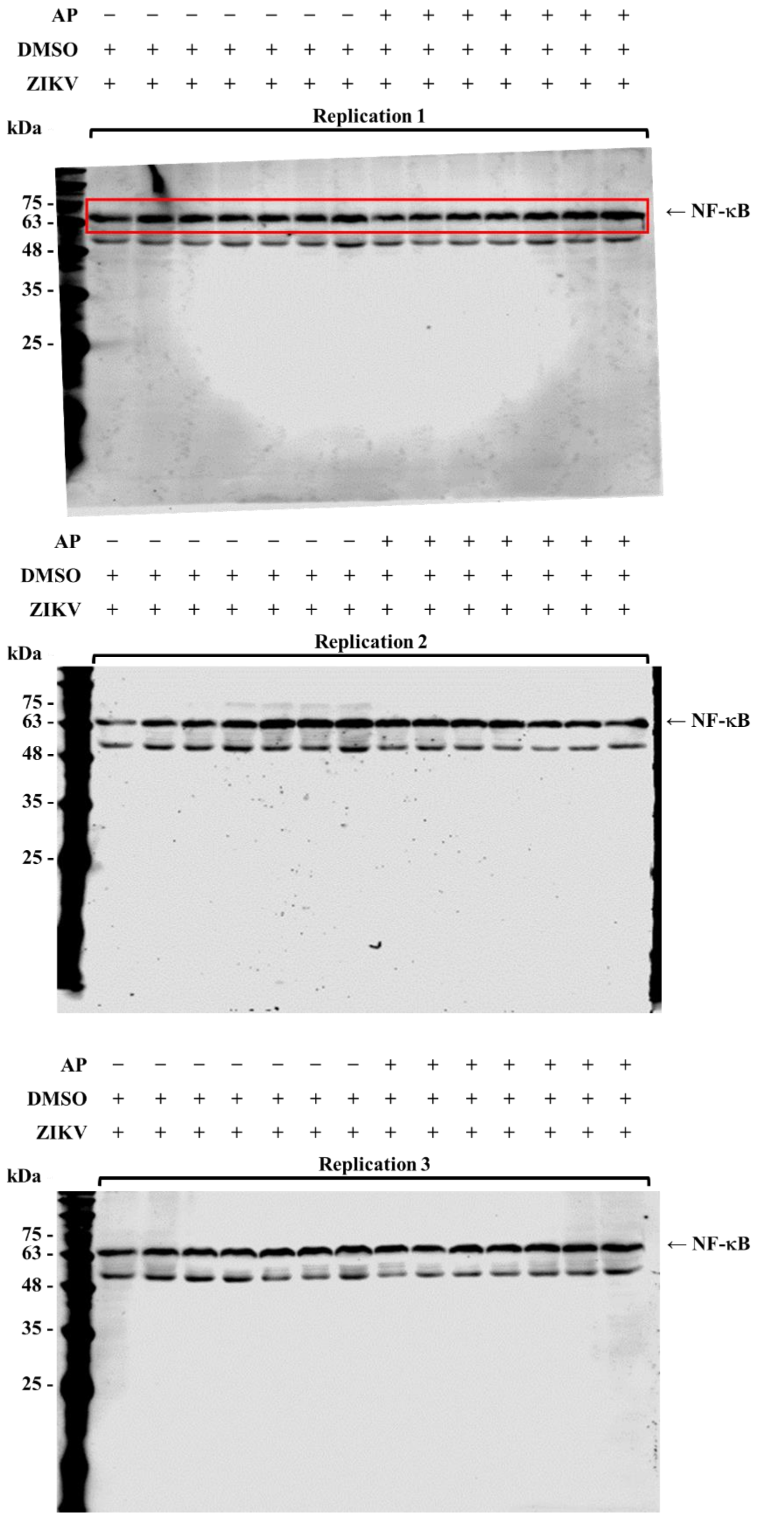

Ratio of Western blot for Figure 6C: Phospho NF-κB (fold change)

Time (h.p.i.)	ZIKV					
	DMSO			AP		
0	1.000	1.000	1.000	0.515	1.393	1.531
1	0.866	0.672	0.322	0.259	0.521	0.710
3	1.146	1.064	1.141	0.648	0.853	2.167
6	0.999	1.031	1.414	0.756	1.085	1.769
12	0.517	1.446	1.754	1.988	2.143	2.868
24	1.826	3.135	3.990	3.612	3.573	5.547
48	2.631	3.725	4.868	3.676	4.345	6.871

Original Western blot for Figure 7B

Immunoblot: p-p38

Red box represents the cropped image used in manuscript figure 7B: p-p38.

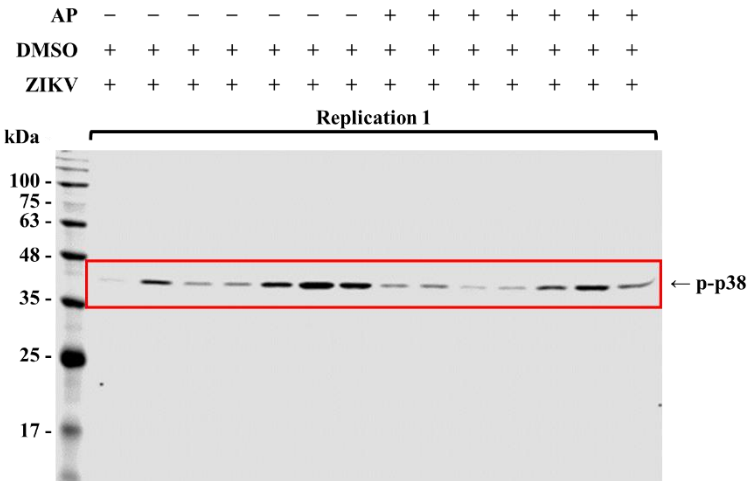

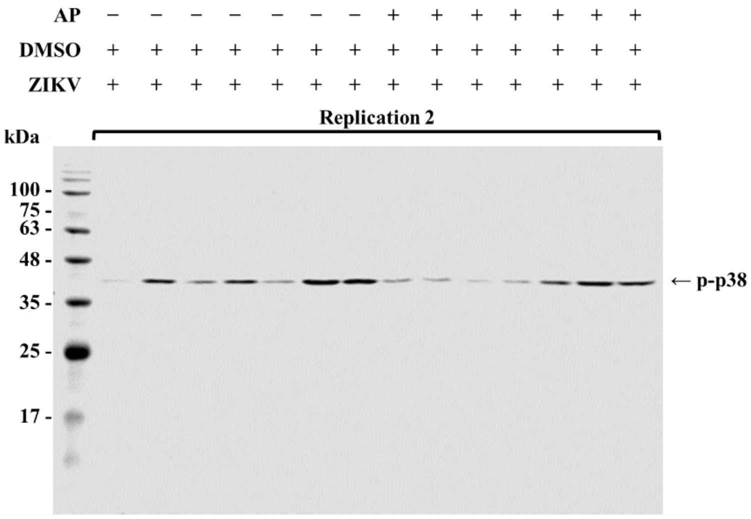

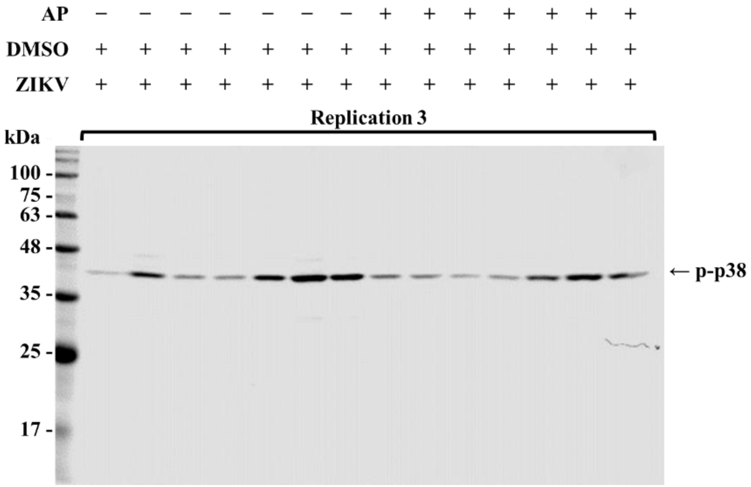

Original Western blot for Figure 7B

Immunoblot: p38

Red box represents the cropped image used in manuscript figure 7B: p38.

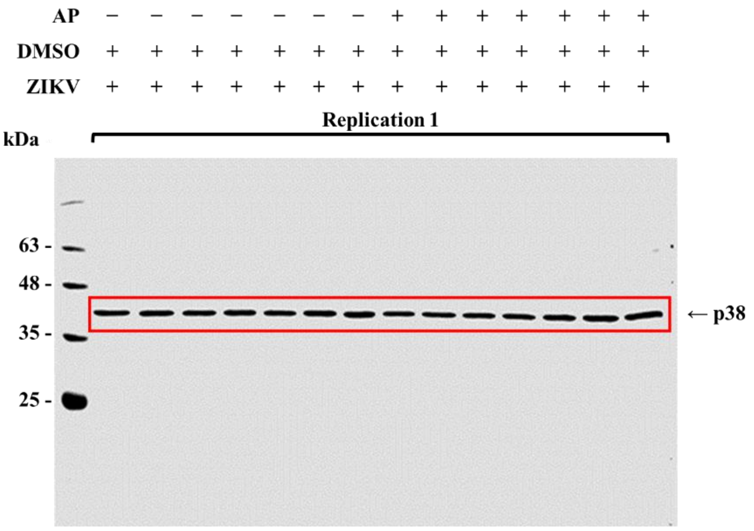

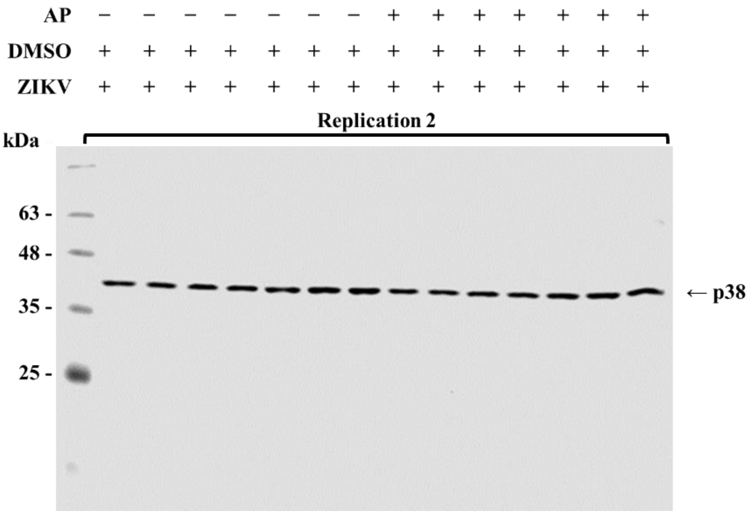

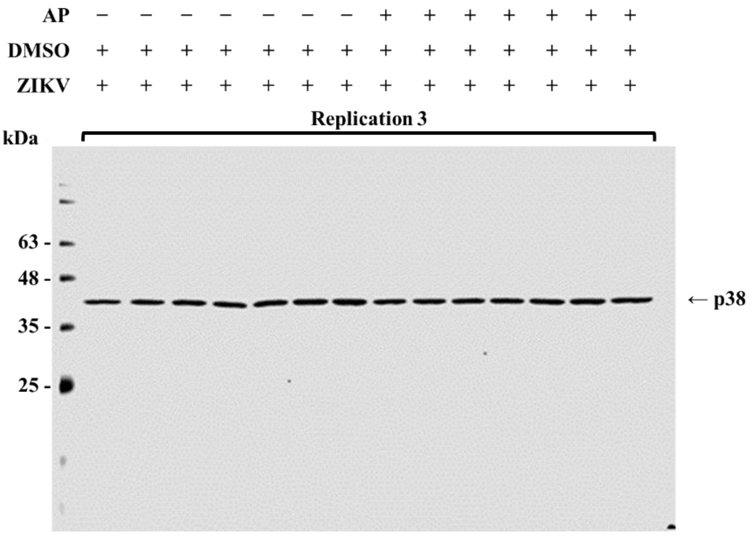

Ratio of Western blot for Figure 7C: Phospho p38 (fold change)

Time (h.p.i.)	ZIKV					
	DMSO			AP		
0	1.00	1.00	1.00	14.18	11.38	12.46
1	22.81	21.89	20.39	5.88	8.81	7.53
3	10.66	11.24	10.52	1.72	3.24	2.68
6	5.48	6.85	5.69	3.48	1.20	2.72
12	25.67	26.78	25.18	19.09	17.70	17.05
24	44.57	48.02	44.20	35.41	32.90	31.30
48	44.10	40.25	40.30	16.88	16.78	16.55

Original Western blot for Figure 8B

Immunoblot: p-ERK1/2

Red box represents the cropped image used in manuscript figure 8B: p-ERK1/2.

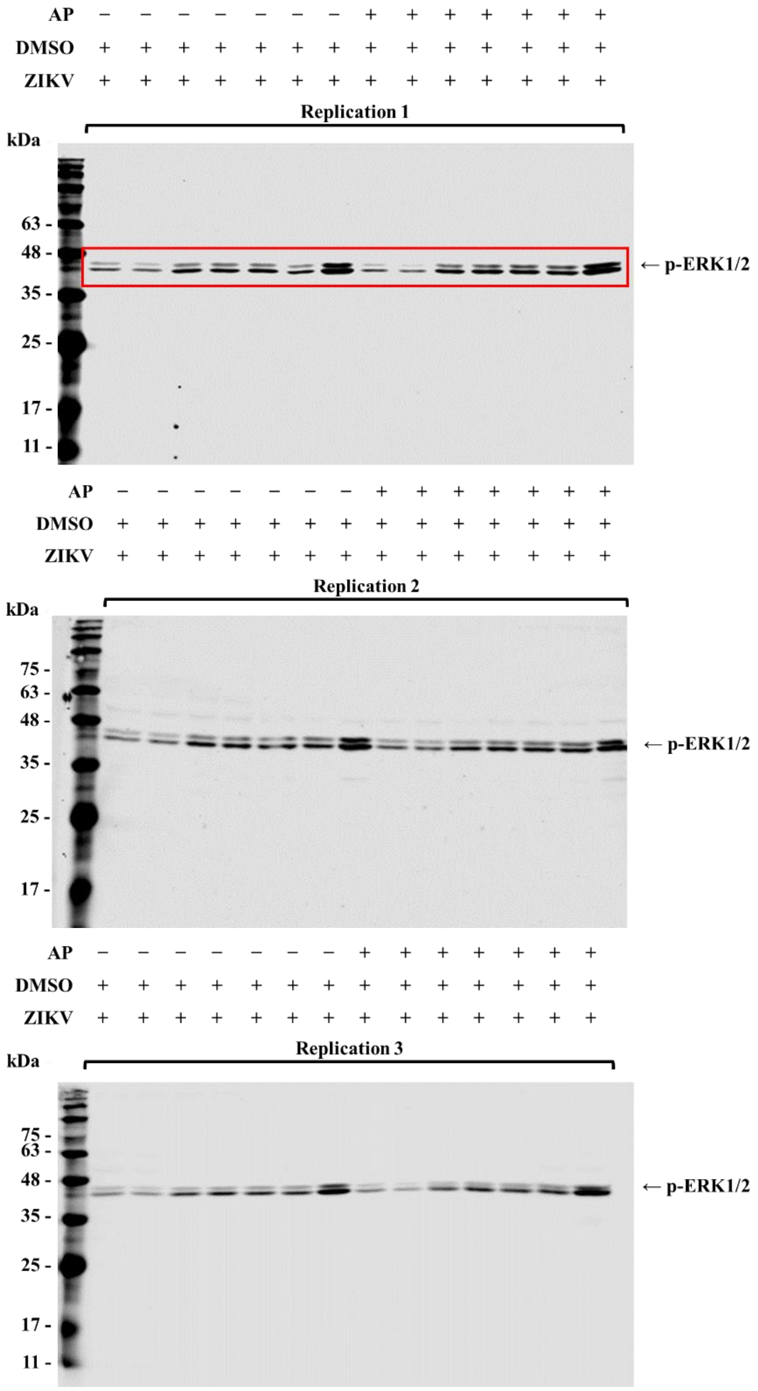

Original Western blot for Figure 8B

Immunoblot: ERK1/2

Red box represents the cropped image used in manuscript figure 8B: ERK1/2.

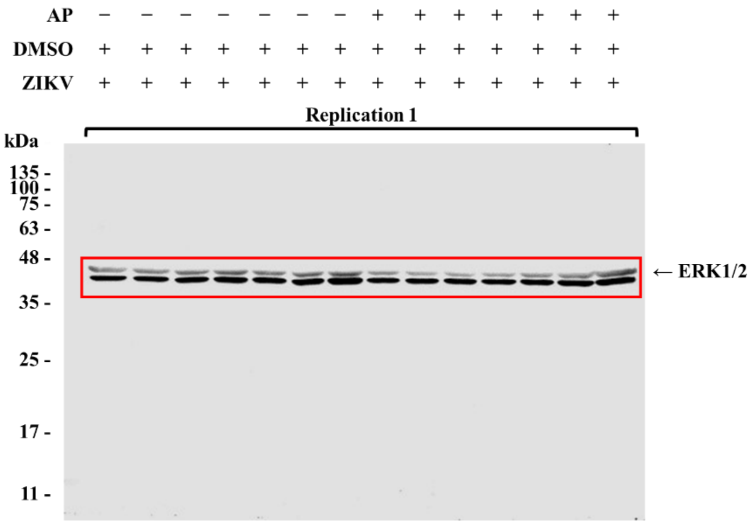

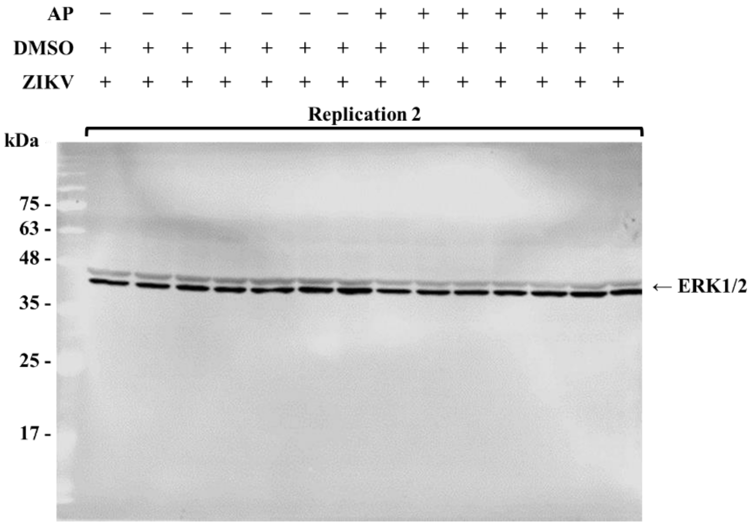

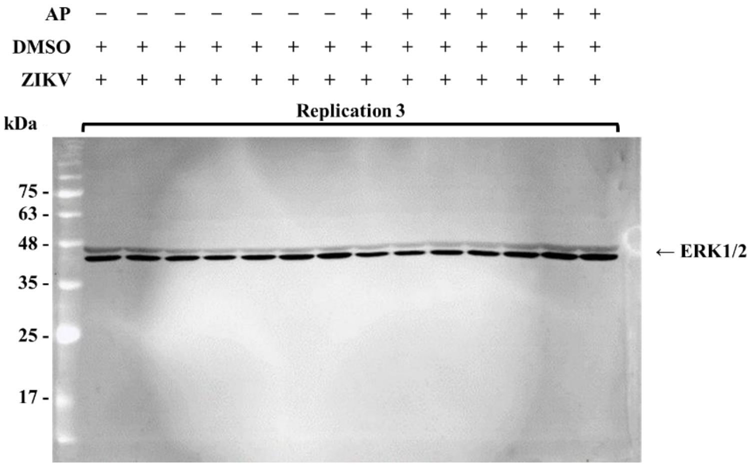

Ratio of Western blot for Figure 8C: Phospho ERK1/2 (fold change)

Time	ZIKV					
(h.p.i.)	DMSO			AP		
0	1.000	1.000	1.000	1.226	1.832	1.737
1	0.586	0.911	0.438	0.509	0.983	0.461
3	1.036	2.003	1.860	1.078	2.034	2.036
6	1.427	2.413	2.305	1.717	2.591	2.997
12	1.437	2.044	2.175	1.856	2.727	3.467
24	1.446	2.717	1.695	2.158	2.914	3.343
48	2.387	4.243	5.112	2.778	3.860	5.769

Original Western blot for Figure 9B

Immunoblot: p-JNK

Red box represents the cropped image used in manuscript figure 9B: p-JNK.

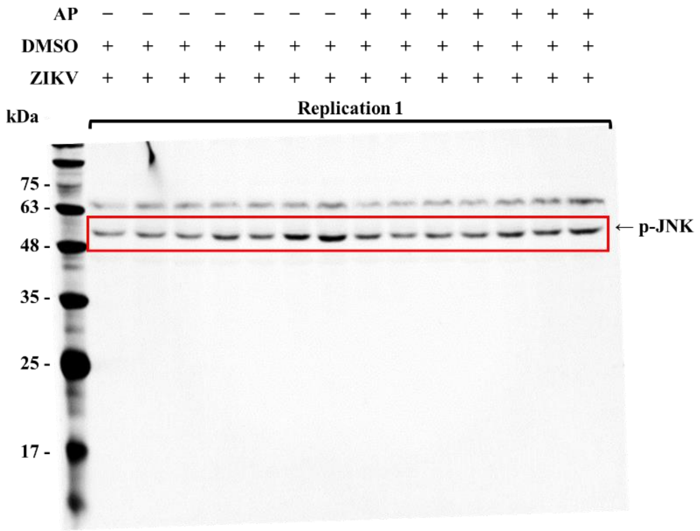

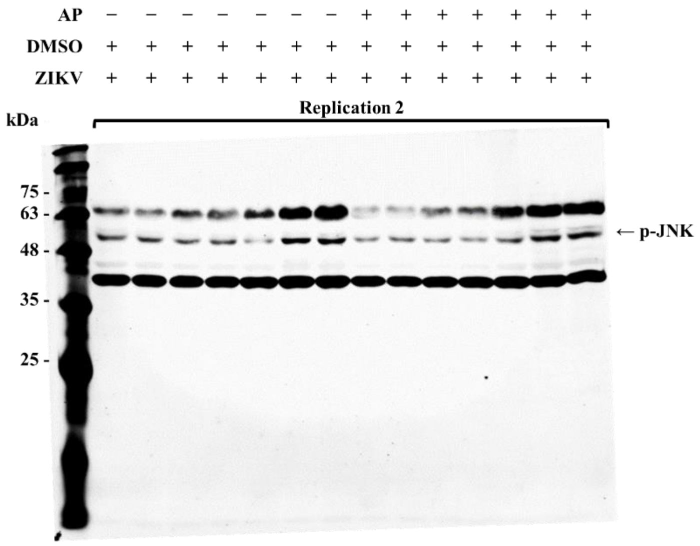

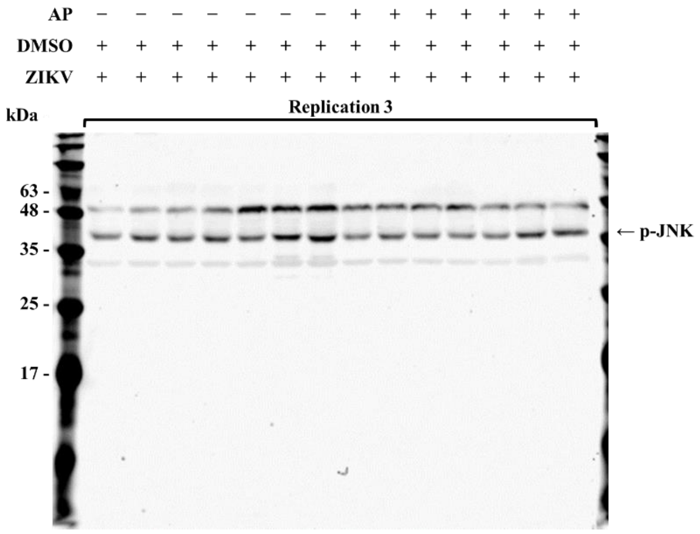

Original Western blot for Figure 9B

Immunoblot: JNK

Red box represents the cropped image used in manuscript figure 9B: JNK.

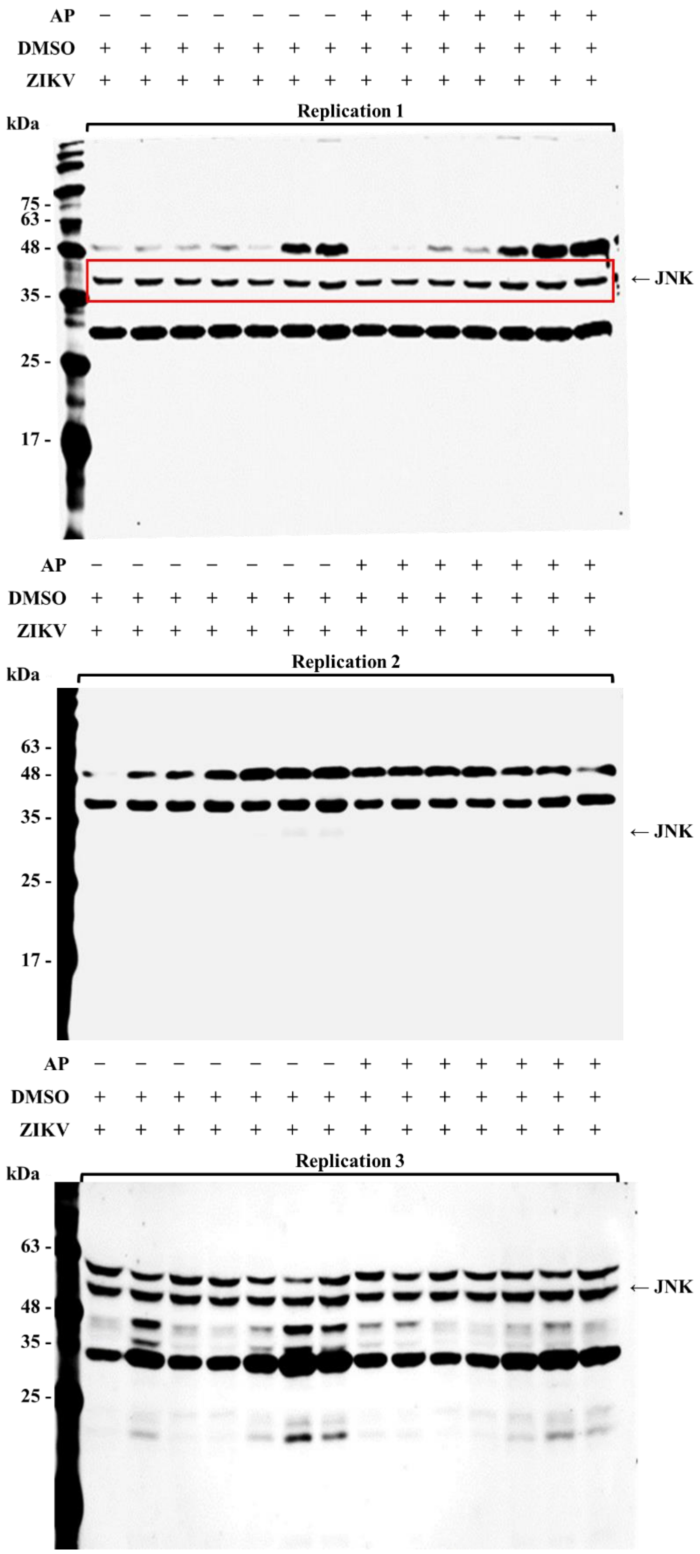

Ratio of Western blot for Figure 9C: Phospho JNK (fold change)

Time	ZIKV					
(h.p.i.)	DMSO			AP		
0	1.000	1.000	1.000	1.963	0.429	1.140
1	1.194	0.794	1.537	1.225	0.398	1.447
3	0.966	0.497	1.490	1.306	0.269	1.089
6	1.707	0.682	1.666	1.287	0.227	1.092
12	1.283	0.215	1.449	1.868	0.716	0.990
24	2.303	1.492	1.956	1.947	1.665	1.763
48	2.917	1.680	2.318	2.404	1.822	1.995

Original Western blot for Figure 6B-9B

Immunoblot: Actin

Red box represents the cropped image used in manuscript figure 6B-9B: Actin.

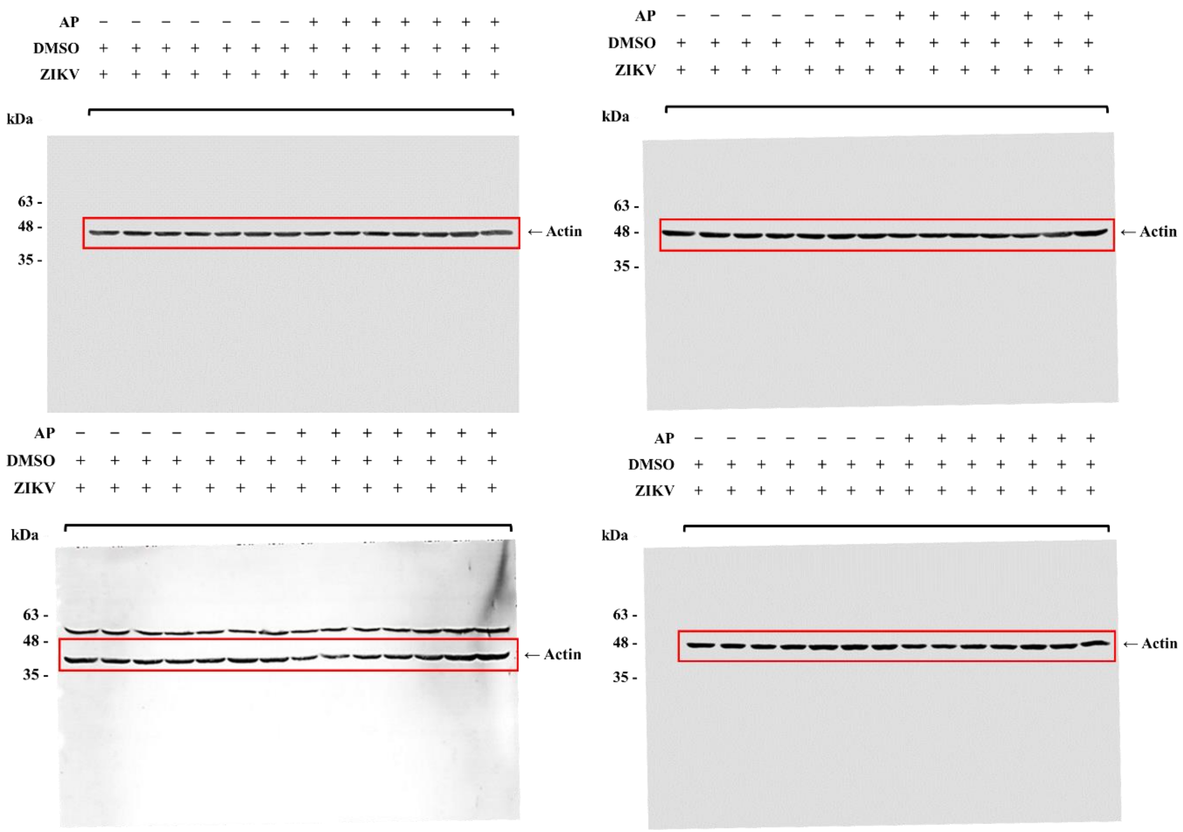

Supplement: Supplementary file 1 [file pharmaceutics-14-02800-s001.zip › pharmaceutics-2042731-supplementary.pdf]
